# Nanofiltration‐Enabled In Situ Solvent and Reagent Recycle for Sustainable Continuous‐Flow Synthesis

**DOI:** 10.1002/cssc.201701120

**Published:** 2017-08-16

**Authors:** Tamas Fodi, Christos Didaskalou, Jozsef Kupai, Gyorgy T. Balogh, Peter Huszthy, Gyorgy Szekely

**Affiliations:** ^1^ School of Chemical Engineering The University of Manchester The Mill, Sackville Street Manchester M13 9PL United Kingdom; ^2^ Department of Organic Chemistry and Technology Budapest University of Technology and Economics Szent Gellert ter 4 Budapest 1117 Hungary; ^3^ Compound Profiling Laboratory, Gedeon Richter Plc. PO Box 27 Budapest 1475 Hungary

**Keywords:** catalytic membrane reactors, continuous processes, flow reactors, membranes, Michael Addition

## Abstract

Solvent usage in the pharmaceutical sector accounts for as much as 90 % of the overall mass during manufacturing processes. Consequently, solvent consumption poses significant costs and environmental burdens. Continuous processing, in particular continuous‐flow reactors, have great potential for the sustainable production of pharmaceuticals but subsequent downstream processing remains challenging. Separation processes for concentrating and purifying chemicals can account for as much as 80 % of the total manufacturing costs. In this work, a nanofiltration unit was coupled to a continuous‐flow rector for in situ solvent and reagent recycling. The nanofiltration unit is straightforward to implement and simple to control during continuous operation. The hybrid process operated continuously over six weeks, recycling about 90 % of the solvent and reagent. Consequently, the E‐factor and the carbon footprint were reduced by 91 % and 19 %, respectively. Moreover, the nanofiltration unit led to a solution of the product eleven times more concentrated than the reaction mixture and increased the purity from 52.4 % to 91.5 %. The boundaries for process conditions were investigated to facilitate implementation of the methodology by the pharmaceutical sector.

## Introduction

Continuous‐flow reactors can provide cost savings, facile automation, a higher level of consistency and certainty, improved overall safety, and a reduced carbon footprint.1a–1c The application of polymer‐supported catalysts in flow reactors have been widely researched in recent years because 1) the use of a polymer support eliminates the need for the removal of the catalyst from the desired product, and 2) reuse is built into the process. However, the downstream processing (i.e. concentration, purification, and isolation) in continuous production remains challenging. Recent efforts demonstrate the need for the development of integrated continuous synthesis– purification processes.2a–2b It has recently been realized that traditional downstream separation processes can account for as much as two thirds of the total manufacturing costs, and eventually contribute half of the industrial energy usage.3a–3b Solvent usage in the pharmaceutical industry accounts for 60 % of the total energy consumption required for the production of active pharmaceutical ingredients.^4^ From another perspective, solvents account for 80–90 % of the overall mass during the manufacturing processes in pharmaceutical industries.^5^ Continuous‐flow reactors can only be truly sustainable if solvent recovery is employed.6a–6b Solvent waste has a large carbon footprint and consequently it is detrimental to the sustainability of the process. Organic solvent nanofiltration (OSN) is a green technology,^7^ that has been used for solvent recovery.8a–8g OSN can be coupled to continuous processes for in situ separations.8g, 9 This approach is in line with the “Roadmap to a resource efficient Europe” instigated by the European Commission, seeking to eliminate waste through deliberate process design with resource efficiency and recycling in mind.^10^

**Figure 1:**
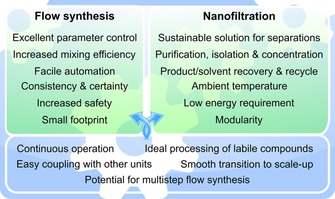
Synergistic relationship between flow synthesis and nanofiltration.

With profit margins growing thin, there is an imperative for minimizing both the cost and environmental impact through process intensification. As effective tools, continuous processing11a,11b and membrane separations3b, 7, 12 have been recognized as core technologies that can enable green process engineering. The newest generation of nanofiltration membranes have stable performance in aggressive media, including extreme pH solutions and organic solvents,^7^, ^13^ which creates new possibilities for continuous solvent recovery, product purification, and concentration. Figure 1 shows the positive attributes of continuous‐flow synthesis and nanofiltration and how they reinforce each other. These technologies offer the opportunity toward smooth integration with further unit operations. However, attempts for the synergistic coupling of flow reactors with nanofiltration are scarce (Table 1 and references therein). In this context nanofiltration was used for the recovery of homogeneous catalysts as well as for solvent exchange. Herein the development of a nanofiltration‐enabled in situ solvent and reagent recycling process to improve the sustainability of flow reactors is presented.

**Table 1:** Continuous hybrid processes comprising of flow reactors and nanofiltration.

Reference	Role of membrane	Membrane	Reaction	Reactor	Solvent	Catalyst
2a	Catalyst retention and solvent exchange	PEEK and Duramem 150	Heck coupling	PFR‐*m*‐CSTR	DMF→EtOH	Pd complex^[a]^
9f	Catalyst removal	Inopor TiO_2_	Suzuki coupling	Coil reactor	EtOH, *i*PrOH	Pd complex^[a]^
						
14	Catalyst recycling	PEEK	Asymmetric hydrogenation	PBR	Toluene, *t*BuOH	Ru complex^[a]^
15	Catalyst retention	PEEK	Heck coupling	PFR‐*m*‐CSTR	DMF	Pd complex^[a]^
						
This work	Solvent and reagent recycling	Duramem 150	Michael Addition	PBR	Acetone	Trialkylamine base^[b]^

[a] Homogeneous. [b] Heterogeneous. PEEK: polyether ether ketone; PFR: plug flow reactor; CSTR: continuous stirred‐tank reactor; PBR: packed bed reactor; ‐*m*‐ refers to well‐mixed DMF: *N*,*N*‐dimethylformamide.

**Scheme 1:**
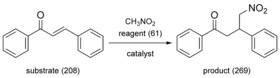
Michael Addition of nitromethane reagent (5 equiv, 60 mm) to chalcone substrate (1 equiv, 12 mm) catalyzed by polymer‐supported trialkylamine base, yielding 4‐nitro‐1,3‐diphenylbutan‐1‐one product. The molecular weights (in g mol^−1^) are given in parentheses for each compound.

Michael Addition of nitromethane to *trans*‐chalcone was selected as a model reaction (Scheme 1), since polymersupported organocatalysts have attracted a great deal of attention in continuous‐flow Michael Addition processes.^16^ Furthermore, OSN was used for homogeneous catalyst recycling in a Michael Addition.^17^ Hodge et al. reported the first application of a weak anion‐exchange resin Amberlyst A21 as a polymer‐supported catalyst to promote Michael Addition in continuous‐flow conditions,1c which inspired the catalyst screening disclosed herein. The most efficient catalysts were tested in a continuous‐flow packed‐bed reactor. Subsequently a nanofiltration membrane unit was connected to the reactor to allow in situ solvent and reagent recycling, and to consequently improve the sustainability of the synthesis.

## Results and Discussion

### Optimization of the flow reactor

Stable, long‐term operation of continuous hybrid processes require an efficient catalyst/solvent system for the chosen Michael Addition. These atom‐efficient reactions are usually promoted by acids, bases, metal salts, or organocatalysts. With application of solid‐base catalysts, formation of undesirable side‐products resulting from polymerization, bis‐addition, and self‐condensation could be prevented and salt formation following the neutralization of soluble bases with acids could be avoided. Amberlyst A21, a polymer‐supported weak anion‐exchange resin featuring a dialkylbenzylamine base was reported to be a cheap, commercial catalyst for Michael Addition reactions.1c The performance of this catalyst was tested in numerous solvents at varying temperatures (Table 2). Using ethyl acetate and acetone solvents resulted in 100 % conversion at 50 °C.

**Table 2:** Batch‐mode conversion [%] as a function of catalyst, solvent, and temperature for the Michael Addition reaction.

		*T* [°C]				
Catalyst	Solvent	30	40	50	60	70
Amberlyst A21	MeOH	8	17	32	51	56
	EtOH	23	34	41	53	61
	CH_3_NO_2_	11	18	20	21	27
	*i*PrOH	42	67	81	92	96
	EtOAc	77	89	100	–	–
	Acetone	78	91	100	–	–
	MeCN	61	76	91	100	–
						
Amberlite IRA67	MeOH	6	15	29	52	61
	EtOH	13	19	37	58	73
	CH_3_NO_2_	7	15	23	32	44
	*i*PrOH	55	78	90	100	–
	EtOAc	71	84	97	100	–
	Acetone	76	87	100	–	–
	MeCN	74	83	96	100	–

**Figure 2:**
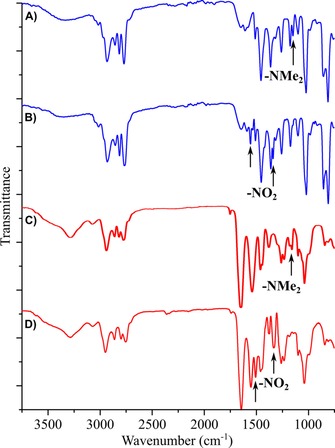
Nitromethane‐induced degradation of the trialkylamine base catalysts: infrared spectra of Amberlyst A21 before (A) and after (B) the Michael Addition at 50 °C, and Amberlite IRA67 before (C) and after (D) the Michael Addition at 60 °C. For (B) and (D), the experiments were carried out in a flow reactor at 1 mL min^−1^ flowrate in acetone for 48 h.

**Figure 3:**
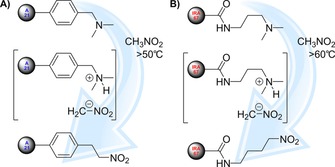
Mechanism of nitromethane‐induced degradation of Amberlyst A21 (A) and Amberlite IRA67 (B) after the Michael Addition at 50 and 60 °C, respectively. See Figure 2 for the corresponding infrared spectra.

A rapid decline in the catalytic activity of Amberlyst A21 was observed during flow reactor conditions. Although 100 % conversion was initially achieved in 9.3 min residence time (representing 42 g L^−1^ h^−1^ space–time yield for the reactor), it decreased below 50 % within two days of operation. Figures 2 and 3 show the IR spectra for the catalyst inactivation process and the underlying mechanism, respectively. The C−N stretching vibrations assigned to aliphatic amine end‐groups of trialkylamine catalyst constituents were observed as medium‐strength bands at 1203 cm^−1^ (Figure 2 A and C). After catalyst inactivation these bands disappeared and the N−O stretching vibrations of aliphatic nitro group were observed at 1576 cm^−1^ (asymmetrical) and 1371 cm^−1^ (symmetrical) as intense bands (Figure 2 B and D). The Michael Addition reaction is promoted by the basic character of the *N*,*N*‐dimethyl *N*‐benzylamino group (p*K*_b_=5.02; conjugated acid p*K*_a_=8.98) of the catalyst and the C−H‐acidic nature of the reagent (p*K*_a_=10.2). The basic tertiary amino group deprotonates nitromethane through an equilibrium reaction that results in the formation of a nitronate intermediate. This ambident nucleophile attacks the soft‐electrophilic center of the substrate. Under mild conditions (low temperature and/or short residence time; low amount of nitromethane) this nucleophilic addition is kinetically favorable according to the hard and soft acid–base (HSAB) principle. However, after longer residence time, the excess of nitromethane can promote a side‐reaction. The nitronate group can attack the electrophilic α‐position of the resin, which results in the formation of a protonated dimethylamino group. This good leaving group can undergo a nucleophilic substitution on a resin functional group (Figure 3). In the case of Amberlyst A21, the benzyl group promotes this reaction, because it uses the π system of the benzene ring in conjugation with the *p* orbital that stabilizes the transition state. On the other hand, Amberlite IRA67 lacks this stabilizing conjugation for the transition state that hinders this side‐reaction. The underlying theory supports the fact that inactivation of the Amberlite IRA67 resin occurred at 60 °C, which is 10 °C higher than the threshold inactivation temperature for the Amberlyst A21 resin.

**Figure 4:**
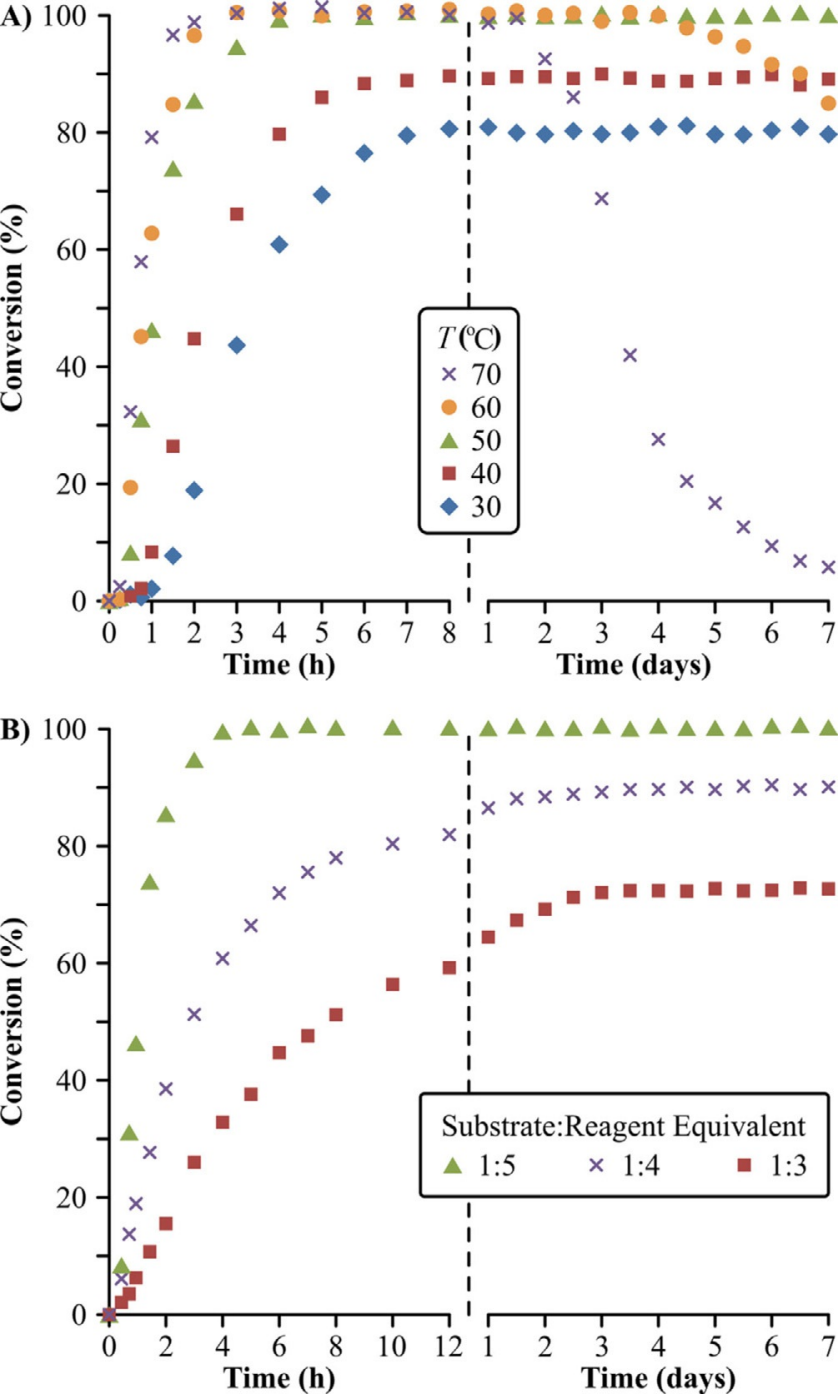
Optimization of reaction conditions for catalysis with Amberlite IRA67: A) temperature dependency for 5 equiv of reagent, and B) effect of reagent excess on the reaction conversion at 50 °C. All experiments were performed at 1 mL min^−1^ flowrate in acetone.

The Amberlite IRA67 catalyst was further characterized in the flow reactor with respect to the effect of temperature and reagent excess on conversion (Figure 4). After 8 h of continuous operation, stable 80 % and 88 % reaction conversion was achieved during seven days of runtime at 30 and 40 °C, respectively. Full conversion was reached at 50–70 °C within 4 h of operation. However, catalyst inactivation was observed within 2–4 days at 60–70 °C. The attained conversion also heavily depends on the amount of reagent employed; 70 %, 90 %, and 100 % conversion was obtained at 50 °C with the use of 3, 4, and 5 equiv of reagent, respectively. Consequently, 50 °C and 5 equiv of reagent were selected for the development of the continuous hybrid process.

**Figure 5:**
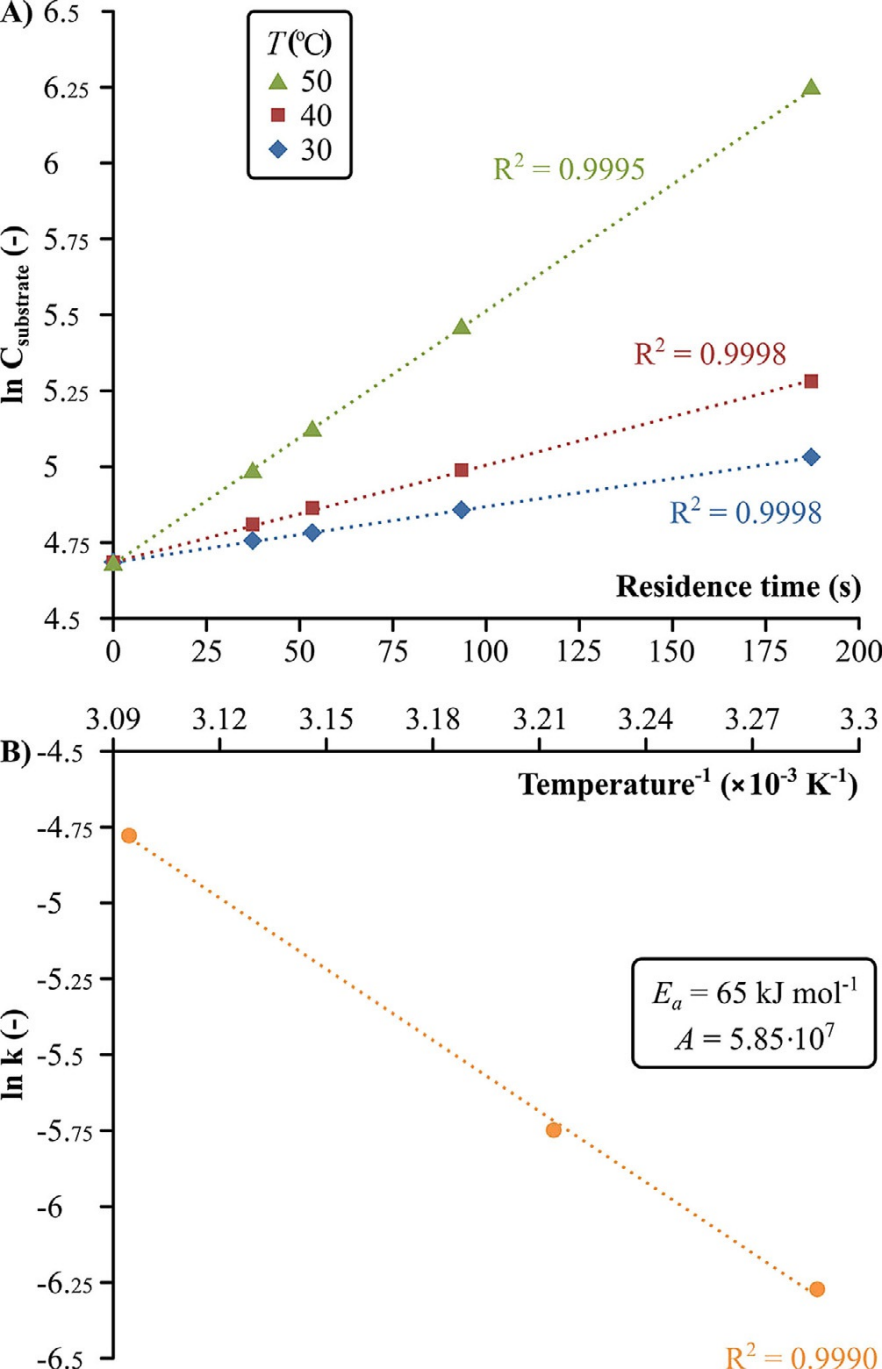
Reaction kinetics for catalysis with Amberlite IRA67: A) the first‐order kinetic plot at various temperatures, and B) the Arrhenius plot and calculated apparent activation energy (*E_a_*) and pre‐exponential factor (*A*) for the Michael Addition.

Since the reagent is applied in high excess and the substrate is the limiting species, the reaction can be assumed to follow pseudo‐first order kinetics.6b Consequently, the total reaction rate is described in Equation (1), and the application of t=0 and $C_{\mathrm{substrate}}=\;C_{\mathrm{substrate}}^{0}$ boundary conditions gives Equation (2).

(1) $$r=-r_{\mathrm{substrate}}=-\frac{{dC}_{\mathrm{substrate}}}{dt}=k^{{}^{'}}C_{\mathrm{substrate}}$$

(2) $$\ln C_{\mathrm{substrate}}=-k^{{}^{'}}t+\ln C_{\mathrm{substrate}}^{0}$$

where *r* is the total reaction rate, *k*′ is the pseudo‐first‐order reaction rate coefficient (in s^−1^), and *C* is the concentration of substrate (in mol L^−1^). The experimental results given in Figure 5 show excellent correlation with the first‐order kinetic model that validate the assumed linear relationship between ${ln(C}_{substrate})$ and t . Refer to the Supporting Information for the conversions at different flowrates. The reaction rate coefficient was determined at different temperatures to estimate the apparent activation energy (*E_a_*) and pre‐exponential factor (*A*) for the reaction (Figure 5 B) using the Arrhenius equation [Equation (3)]:

(3) $$k{}^{'}=Ae^{-\frac{E_{a}}{RT}}$$

where *R* is the ideal gas constant of 8.314 J K^−1^ mol^−1^ and *T* is the temperature in K. The apparent activation energy was found to be 65 kJ mol^−1^, which falls within the 19–76 kJ mol^−1^ range reported for the same Michael Addition catalyzed by cinchona‐urea‐based organocatalysts.^18^ The obtained kinetic parameters can be used for process modelling toward automation.

### Membrane screening for the optimization of the separation

**Figure 6:**
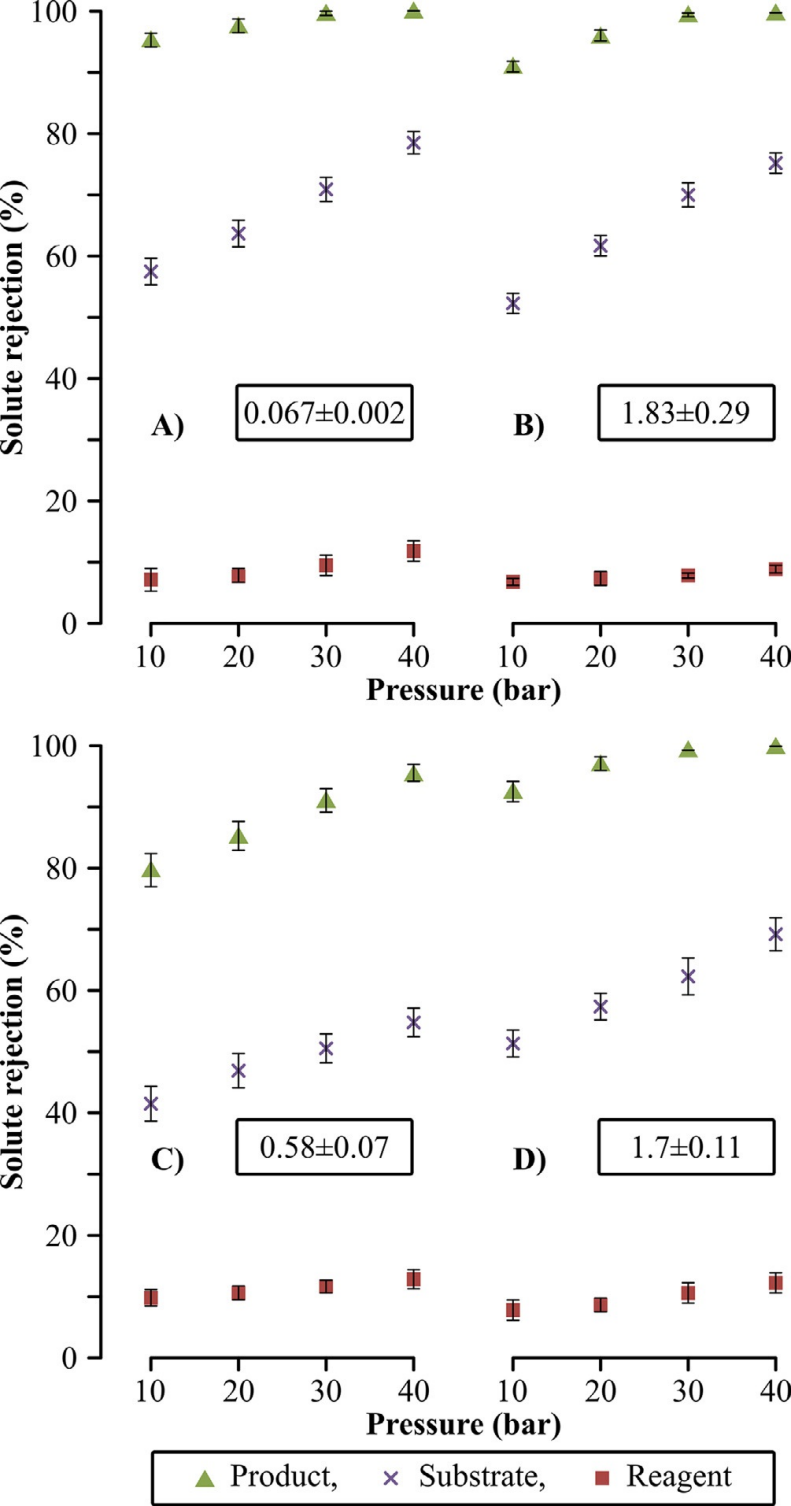
Rejection profiles for Duramem 150 (A), GMT‐oNF‐2 (B), SolSep NF030705 (C), and PBI (D) membranes at 10–40 bar pressure. The feed solution comprised of the product, the substrate, and the reagent each at 1 g L^−1^ concentration in acetone. The permeance values expressed in L m^−2^ h^−1^ bar^−1^ are given within the boxes. Refer to the Supporting Information for the membrane screening process scheme.

The nanofiltration unit coupled to the flow reactor has a two‐fold role: 1) recycle the solvent and the reagent, and simultaneously 2) concentrate the product during continuous operation. To this effect, Duramem 150, GMT‐oNF‐2, SolSep NF030705, and polybenzimidazole (PBI) solvent‐resistant nanofiltration membranes were screened at 10–40 bar pressure (Figure 6).

Solute rejections [Equation (4)] were obtained taking into account the retentate and permeate concentrations. Permeance [Equation (5)] showing the volume of liquid that permeates the membrane (*V_permeate_*) per membrane area (*A_m_*), per unit of time (*t*), and per applied pressure (*p*) was also measured. High product rejection prevents undesired recycling back into the reactor and subsequent accumulation, which can lead to decreases in productivity. On the contrary, low reagent rejection is needed to 1) minimize product contamination in the retentate stream leading to higher product purity, and 2) minimize waste generation by maximizing the reuse of the reagent. Duramem 150 membrane operating at 40 bar pressure was selected for the continuous process because these conditions exhibited the highest rejection for the product (100 %), but still maintaining low rejection for the reagent (12.2 %).

(4) $$\mathrm{Solute}\;\mathrm{rejection}\;[\%]=100\times \left(1-\frac{C_{\mathrm{permeate}}}{C_{\mathrm{retentate}}}\right)$$

(5) $$\mathrm{Permeance}\;[L\;m^{-2}\;h^{-1}\;{\mathrm{bar}}^{-1}]\;=\;\frac{V_{\mathrm{permeate}}}{A_{m}\;\times \;t\;\times \;p}$$

### Continuous‐flow reactor/nanofiltration hybrid process

**Figure 7:**
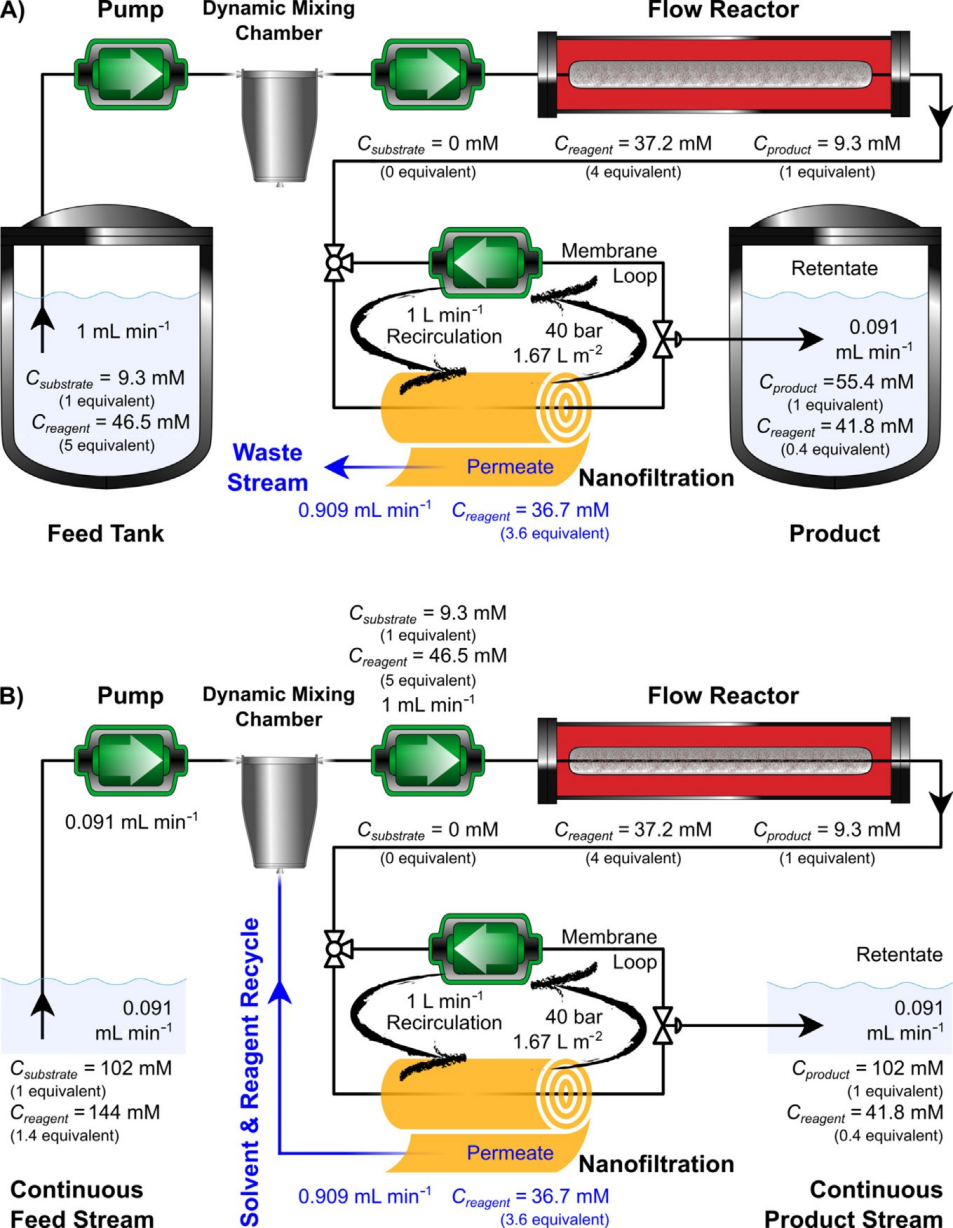
Process configuration and conditions for the start‐up (A) and continuous steady‐state (B) operation of the hybrid process. The jacketed flow reactor operated at 50 °C allowing 100 % conversion of the substrate. The nanofiltration unit was operated continuously at 40 bar, splitting the crude mixture into a concentrated, product‐rich retentate stream, and a reagent‐rich permeate stream. Initially the permeate stream was discarded (A) and once steady‐state was reached, recycling of the permeate stream was commenced (B), and the feed concentration and flowrate were adjusted.

The hybrid process comprised of a flow reactor and a nanofiltration unit that were filled with acetone at the start of continuous operation (Figure 7). The feed solution containing 9.3 mm substrate and 5 equiv of reagent in acetone was allowed to pass through the packed‐bed flow reactor loaded with 8.2 g of catalyst. The flowrate was set to 1 mL min^−1^ allowing 100 % substrate conversion after 6.25 min residence time. Consequently, the crude product stream comprised of 1 equiv of product and 4 equiv of reagent. This stream was fed into the nanofiltration unit comprising 0.021 m^2^ membrane area and 35 mL volume. A recirculation pump set at 1 L min^−1^ ensured homogeneous solute concentration in the membrane loop. The membrane split the crude mixture into a concentrated, product‐rich retentate stream (10 % of feed flowrate), and a reagent‐rich permeate stream (90 % of feed flowrate). During start‐up of the continuous system the permeate stream was discarded (Figure 7 A). Once steady‐state permeate concentration was reached, recycling of the permeate stream through a dynamic mixing chamber was commenced (Figure 7 B). To compensate the changes (i.e. concentration and flowrate) induced by the permeate recycle, both the flowrate and concentrations of the feed were adjusted accordingly, and subsequently steady‐state operation was maintained. The new feed flowrate was set to 0.091 mL min^−1^, whereas the feed concentrations were changed to 102 mm substrate and 1.4 equiv reagent.

**Figure 8:**
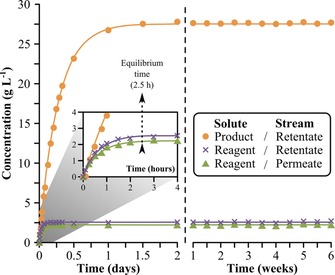
Concentration profile in the nanofiltration unit. The curves show the simulated values and the symbols show the experimental data. The system was operated in the start‐up process configuration (Figure 7 A) until steady‐state concentration was reached (at 2.5 h) for the reagent in the permeate stream. At that point recycling of the permeate stream was commenced.

The concentration profiles of the solutes in the retentate and permeate streams are shown in Figure 8. The experimental data shows good correlation with the predicted curves. Refer to the Supporting Information for the detailed mathematical framework describing the process modelling. The equilibrium time is defined as the time needed to reach 98 % of the steady‐state concentration of a solute, and it was found to be 2.5 h for the reagent in the permeate stream. This is the threshold to start recycling the solvent and the reagent. Until 2.5 h the permeate was discarded (Figure 7 A) and afterward it was recycled back to the flow reactor through a dynamic mixing chamber (Figure 7 B). The product leaves the flow reactor and enters the nanofiltration unit at 2.5 g L^−1^ concentration, and then gets concentrated to 27.5 g L^−1^, hence the considerably longer equilibrium time of 25 h. The hybrid process showed stable performance over six weeks of continuous operation, achieving catalyst turnover number (TON) of 32, catalyst turnover frequency (TOF) of 0.032 h^−1^, productivity of 3.75 g L^−1^ h^−1^ and 91.5 % product purity. It should be noted that the membrane performance could change and fouling could occur over time, which can be mitigated by operating well below the solubility limit of the solutes.^19^ During the six weeks of operation neither catalyst deactivation nor membrane fouling were observed.

**Figure 9:**
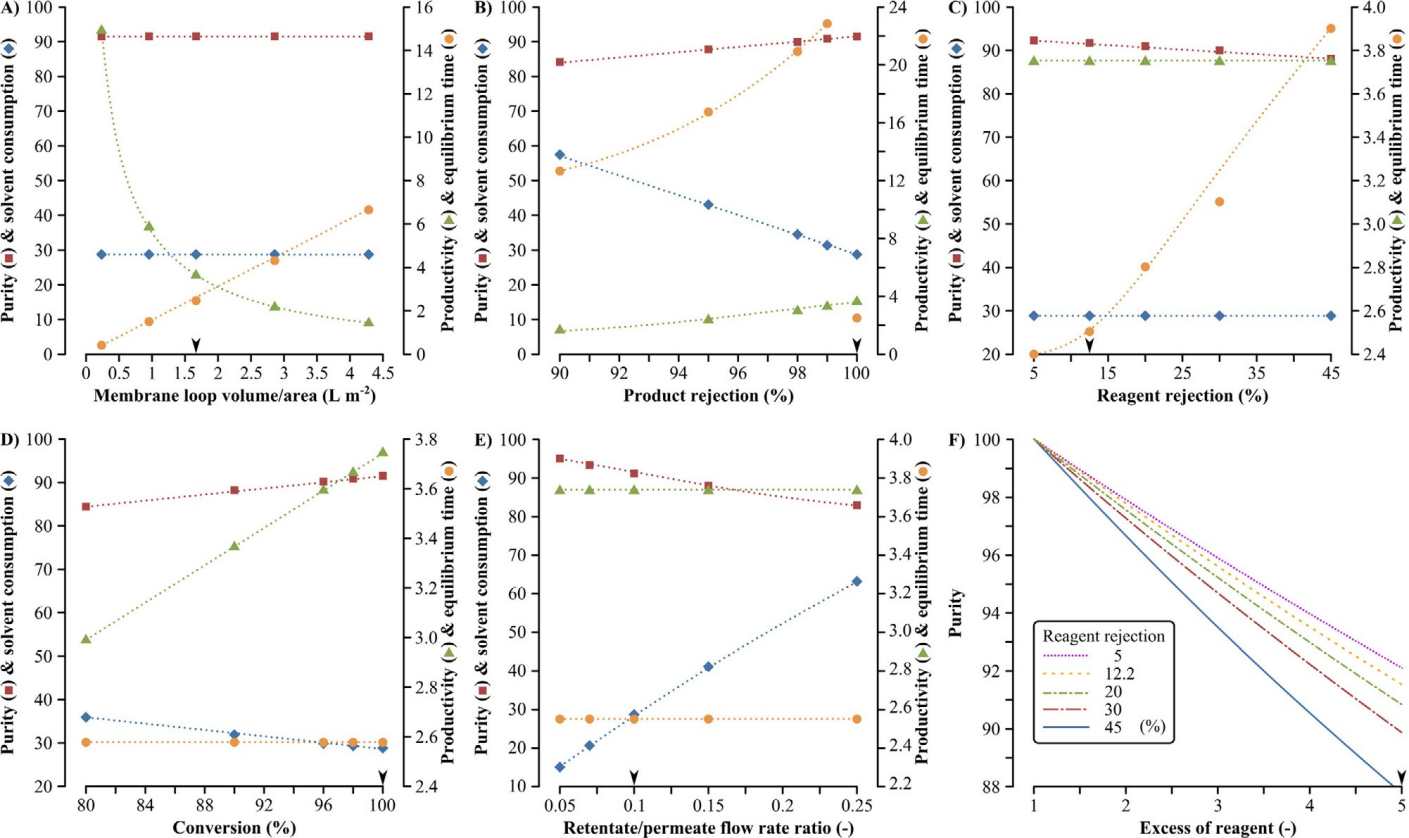
Sensitivity analysis. The variation of six system parameter settings, namely membrane loop volume/area (A), product rejection (B), reagent rejection (C), conversion (D), retentate/permeate flowrate ratio (E), and molar equivalents of the excess of reagent (F), and their effects on four process metrics, namely product purity (▪ [%]), solvent consumption (⧫ [kg kg^−1^]), productivity (▴ [g L^−1^ h^−1^]) and equilibrium time (• [h]). The arrowheads on the *x*‐axes indicate the actual parameter value used for the continuous hybrid process presented in Figure 7 B and Figure 8. For the sensitivity analysis of each parameter, the remaining parameters were kept constant using the same value as for the continuous hybrid process. The excess of reagent (F) influences only the purity process metric.

The effects of various system parameters—namely conversion, product and reagent rejections, retentate/permeate flowrate ratio, and membrane loop volume/area—on solvent consumption, product purity, productivity, and equilibrium time were investigated (Figure 9). The membrane loop volume/area does not affect the solvent consumption and the product purity but its decrease results in the improvement of both productivity and equilibrium time. Although minimization of this volume is practically difficult using flat‐sheet membranes (laboratory scale), the membrane modules used in industrial applications have relatively low values of 0.8–1.2 L m^−2^.^20^ The increase in product rejection is beneficial for all process metrics. On the contrary, the decrease in reagent rejection enhances product purity and shortens equilibrium time, but neither solvent consumption nor productivity is affected. Similar to product rejection, the higher the conversion, the better the process metrics become except for the equilibrium time, which remains constant. Decreasing the retentate/permeate flowrate ratio results in a favorable increase in purity and decrease in solvent consumption whereas the productivity and equilibrium time are not affected. Figure 9 F shows that high‐purity product can be obtained by minimizing the excess of the reagent and the reagent rejection. Furthermore, the lower the excess of reagent, the less influence the reagent rejection has on the product purity. Refer to the Supporting Information for the detailed sensitivity analysis.

### Sustainability assessment of the hybrid process

The effect of nanofiltration‐assisted in situ solvent and reagent recycling on the sustainability of the continuous‐flow synthesis was assessed. Changing from batch to continuous processing in a flow synthesis process can considerably reduce the environmental burden of chemical manufacturing.21a–21d Nonetheless, the use of organic solvents was identified as one of the largest contributor to waste generation in flow synthesis.21a, 22a–22b The E‐factor and carbon footprint, defined in Equation (6) and Equation (7), have pivotal roles in improving resource efficiency and waste minimization in the chemical industries.^10^

(6) $$E-\mathrm{factor}=\frac{\mathrm{kg}\;\mathrm{waste}\;\mathrm{generated}}{\mathrm{kg}\;\mathrm{isolated}\;\mathrm{product}}$$

(7) $$\mathrm{Carbon}\;\mathrm{footprint}=\frac{\mathrm{equivalent}\;\mathrm{kg}\;\mathrm{of}\;{\mathrm{CO}}_{2}}{\mathrm{kg}\;\mathrm{isolated}\;\mathrm{product}}$$

**Figure 10:**
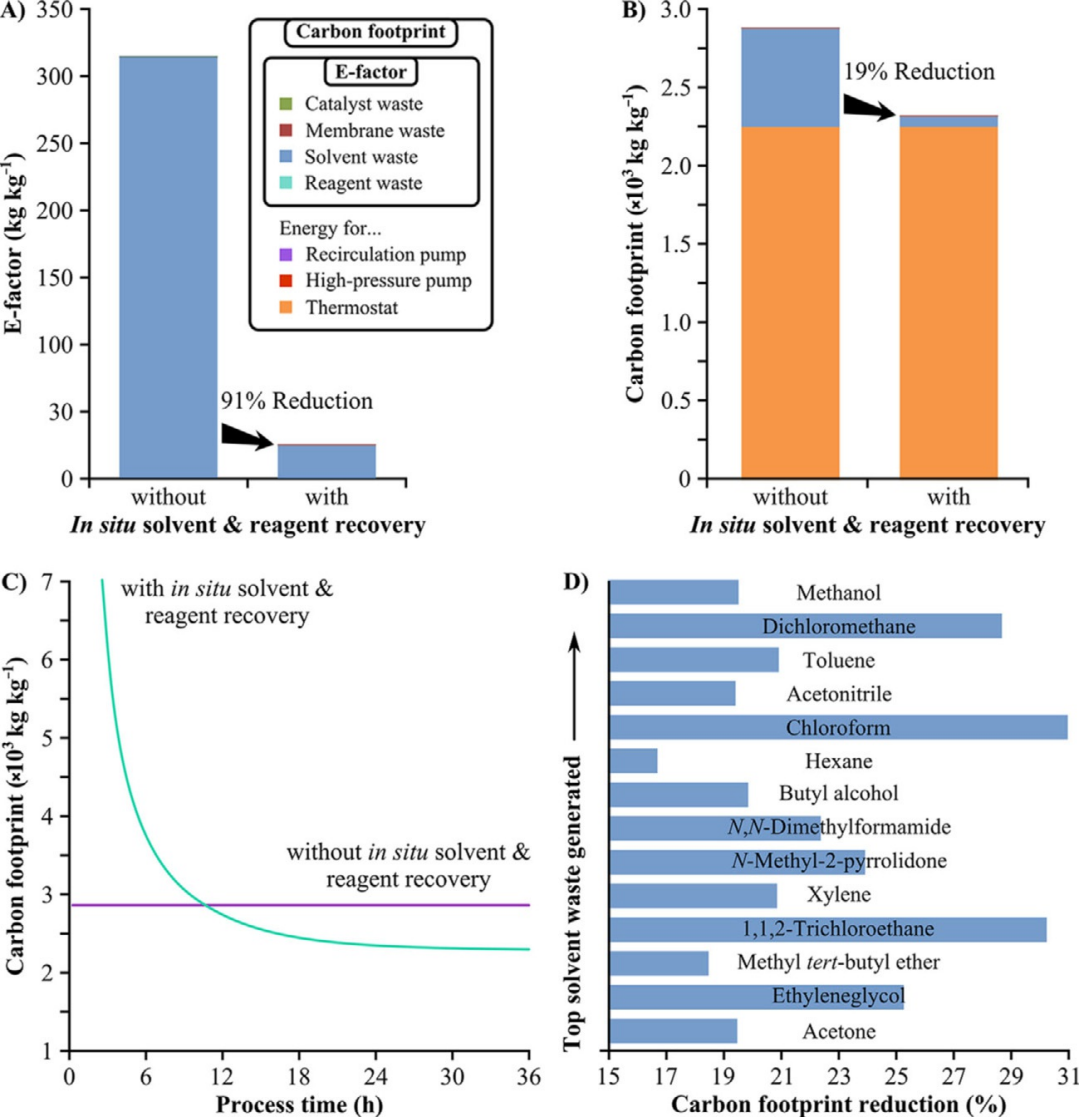
The effect of in situ solvent and reagent recycling on the E‐factor (A) and the carbon footprint (B, C) of the continuous‐flow synthesis. The main contributors of the environmental burden are the solvent waste and the energy to operate the thermostat for the jacket of the flow reactor. The rest of the contributors have cumulative contributions lower than 0.5 %. Refer to the Supporting Information for the actual values for all contributors. Based on the acetone solvent recovery flowrate and the density of the different solvents, the potential of nanofiltration for the carbon footprint reduction can be estimated^25^ for the most common solvent wastes^5^ generated in the pharmaceutical sector (D).

Calculation of the E‐factor ignores any recycled streams and reused reactants or reagents as they are not considered as waste. On the other hand, it neglects the energy required for the process. Consequently, carbon footprint, taking into account waste generation and energy consumption, was also derived to obtain a holistic view of the environmental burden of the hybrid process (Figure 10). Coupling of the nanofiltration unit to the flow reactor allows the recycling of 90.9 % of solvent and 90 % of reagent, corresponding to 287 kg solvent and 0.814 kg reagent per kg of product, respectively (Figure 10 A). The solvent consumption is the main contributor to the E‐factor, and the nanofiltration unit reduces waste generation and raw material usage by 90.7 %. The application of the nanofiltration unit is sustainable,^7^ and in this particular case it poses only 0.4 % of the carbon footprint taking into account both the energy requirements for the pumps and the disposal cost of the membranes. Refer to the Supporting Information for the breakdown of the waste generation and energy consumption.

The carbon footprint contribution of the thermostat for the jacket of the flow reactor is 2257 kg kg^−1^ accounting for 78 % and 97 % of the total carbon footprint in the absence and presence of the nanofiltration unit, respectively (Figure 10 B). In line with the 6th principle of green chemistry,^23^ such a high contribution calls for energy integration within a manufacturing plant. The obtained values for the E‐factor demonstrate that in situ solvent and reagent recycling can transform the process from the non‐green category to the green category based on the industrial classification by Sheldon.^24^ The carbon footprint of the continuous hybrid process rapidly decreases over time and reaches a constant value of 2323 kg kg^−1^ within less than two days of operation, and it takes 10 h to become more environmentally benign than the benchmark process (Figure 10 C). The reduction in carbon footprint depends on the solvent used in the process (Figure 10 D). The potential reduction in the carbon footprint of nanofiltration was assessed considering the top solvent wastes^5^ generated in the pharmaceutical sector. Depending on the carbon footprint potential of each solvent,^25^ the implementation of a nanofiltration unit could result in 17–31 % overall reduction.

The proposed process configuration enables further process‐window expansion. First, the continuous reaction‐separation methodology can be used for sought‐after continuous flow multi‐step organic syntheses.^2^, ^26^ Second, the pressurized system allows the reaction to be realized at elevated temperatures up to supercritical conditions which is of recent interest.^27^

## Conclusions

Increasing demand for more sustainable manufacturing has led to an increasing interest in the area of continuous processing within the pharmaceutical sector. Flow synthesis allows continuous production of fine chemicals in a safe, compact, and controlled environment but subsequent downstream processing remains challenging. Concentration and purification of the product is usually done in batch operation, and these separation processes can account for as much as 80 % of the total manufacturing costs. In this work, a continuous hybrid process comprising of a flow reactor and a subsequent nanofiltration unit for in situ solvent and reagent recycling has been developed. The nanofiltration unit is straightforward to implement and control during continuous operation. A predictive in silico tool—where the main input parameters are the membrane permeance and solute rejections—was developed to understand the boundaries of process conditions. The hybrid process was operated continuously over six weeks, leading to the recovery of about 90 % of the solvent and the excess of the reagent. Consequently, the E‐factor and the carbon footprint were reduced by 91 % and 19 %, respectively. The nanofiltration unit concentrated the product by 11 times and increased the purity from 52.4 % to 91.5 %. In‐line coupling of nanofiltration to flow reactors in continuous operation can significantly improve the sustainability of flow synthesis through in situ solvent and reagent recycling, as well as improving the concentration and purity of the product. The proposed process configuration enables further process‐window expansion for continuous‐flow multi‐step organic syntheses.

## Experimental Section

### Materials and general methods

Chemicals (reagent grade) and solvents (analytical grade) were purchased from Sigma–Aldrich (UK) and were used without further purification. Three commercially available OSN membranes were used: SolSep NF010206, GMT‐oNF‐2 and DuraMem150 (DM150) from SolSep BV, Borsig Membrane Technology GmbH and Evonik‐MET, respectively. 26 wt % polybenzimidazole (MW=27 000 g mol^−1^) containing 1.5 wt % lithium chloride stabilizer dissolved in *N*,*N*‐dimethylacetamide (DMAc) solution was purchased from PBI Performance Products Inc. (USA). Non‐woven polypropylene fabric Novatexx 2471 was sourced from Freudenberg Filtration Technologies (Germany).

^1^H and ^13^C NMR spectra were recorded on a Bruker AV‐400 spectrometer. HPLC measurements were carried out on a VWR HPLC system equipped with a VWR 5160 pump, a VWR 5260 autosampler and thermostat, a VWR 5430 diode array detector (DAD) and an ACE 5 C18 150×4.6 mm, 5 μm column. The pump flowrate was set at 1 mL min^−1^ and the column temperature was 25 °C. The mobile phase was a 1:1 mixture of acetonitrile and water with 0.1 % trifluoroacetic acid. Nitromethane was quantified using a Varian CP‐3800 gas chromatograph (GC) fitted with a Varian Saturn 2200 mass spectrometer (MS) and a Varian VF‐5 ms capillary column (30 m×0.25 mm, 0.25 μm). The GC oven temperature was 27 °C for 4 min, then increased at 15 °C min^−1^ to 200 °C. LCMS measurements were carried out on an Agilent 1100 HPLC equipped with gradient pump, autosampler and photodiode array (PDA) detector. A triple quadrupole mass spectrometer with positive electrospray ionization source was employed as the MS detector. Infrared spectra were recorded on a Bruker Alpha‐T FTIR spectrometer.

### Batch reactions

In a round‐bottomed flask equipped with a reflux condenser, a solution of *trans*‐chalcone (50 mg, 4.8 mm) and nitromethane (5 equiv, 24 mm) was stirred in either methanol, ethanol, 2propanol, ethyl acetate, acetone, or acetonitrile solvents (50 mL). In the case of nitromethane as both solvent and reagent, *trans*‐chalcone (50 mg, 4.8 mm) was directly dissolved in nitromethane (50 mL). To these solutions Amberlyst A21 or Amberlite IRA67 (50 mg) catalyst was added. After stirring the reaction mixture at 30–70 °C temperature for 24 h, conversions were determined by HPLC.

### Continuous‐flow reactions

A Supelco stainless steel column of 10 mm×250 mm dimension was loaded with a solid base Amberlyst A21 (13.1 g) free‐base form or Amberlite IRA67 (8.1 g) free‐base form, respectively. Prior to use the filled column was washed with the reaction solvent (50 mL) with a flowrate of 1.0 mL min^−1^ to remove air and unwanted materials from the surface of the solid base. A feed solution containing *trans*‐chalcone (12 mm) and nitromethane (1–5 equiv, 12–60 mm) was pumped through the column at 1–10 mL min^−1^ flowrate corresponding to 6.25–0.625 min residence time at 30–70 °C temperature. A Lauda Alpha RA12 thermostat was used to control the temperature with ±0.1 °C accuracy in the jacketed flow reactor. The energy consumption of the equipment was measured with a Fluke 1736 power logger with resolution 10 mA and accuracy ±0.1 %.

### Membrane screening

The polybenzimidazole (PBI) membrane was prepared according to our previously reported protocol.8f The feed solution for the membrane screening comprised of a mixture of substrate, reagent, and product, each at 1 g L^−1^ concentration in acetone. The pressure range for the screening was 10–40 bar (1 bar=0.1 MPa) and the tests were carried out in duplicate in a cross‐flow nanofiltration rig.8f The feed solution was recirculated for 24 h followed by collection of samples from the permeate and the retentate streams. The solute rejection values for the substrate and product were determined by LCMS, whereas GCMS was used for the reagent. Refer to the Supporting Information for the membrane screening process scheme.

## Conflict of interest

*The authors declare no conflict of interest*.

## Supporting information

As a service to our authors and readers, this journal provides supporting information supplied by the authors. Such materials are peer reviewed and may be re‐organized for online delivery, but are not copy‐edited or typeset. Technical support issues arising from supporting information (other than missing files) should be addressed to the authors.

Supplementary
