# Supplementary material for: Nanofiltration‐Enabled In Situ Solvent and Reagent Recycle for Sustainable Continuous‐Flow Synthesis
Source: ChemSusChem. 2017 Aug 16;10(17):3435–44. doi: 10.1002/cssc.201701120 (PMC6032941; doi:10.1002/cssc.201701120)
Supplement: Supplementary file 1 — Supplementary [file CSSC-10-3435-s001.pdf]

CHEMISTRY & SUSTAINABILITY
CHEMSUSCHEM
ENERGY & MATERIALS

Supporting Information

Nanofiltration-Enabled In Situ Solvent and Reagent Recycle for Sustainable Continuous-Flow Synthesis

Tamas Fodi+,[a, b, c] Christos Didaskalou+,[a] Jozsef Kupai,[b] Gyorgy T. Balogh,[c] Peter Huszthy,[b] and Gyorgy Szekely*[a]

cssc_201701120_sm_miscellaneous_information.pdf

Supporting Information

for

Nanofiltration-enabled *in situ* solvent and reagent recycle for sustainable continuous-flow synthesis

Tamas Fodi,†[a-c] Christos Didaskalou,†[a] Jozsef Kupai,[b] Gyorgy T. Balogh,[c] Peter Huszthy,[b] and Gyorgy Szekely*[a]

[a] School of Chemical Engineering & Analytical Science, The University of Manchester, The Mill, Sackville Street, Manchester, M13 9PL, United Kingdom

[b] Department of Organic Chemistry and Technology, Budapest University of Technology and Economics, H-1521 Budapest, PO Box 91, Hungary

[c] Compound Profiling Laboratory, Chemical Works of Gedeon Richter Plc., PO Box 27, H-1475 Budapest, Hungary

* gyorgy.szekely@manchester.ac.uk, +44(0)161 306-4366

† These authors equally contributed.

Table of Contents

1. Definitions S2

2. Reaction Kinetics in the Flow Reactor S6

3. Process Configuration for Membrane Screening S7

4. Process Modelling S8

5. Sensitivity Analysis for the Hybrid Process S10

6. Green Metrics Analysis S17

7. Chemical Characterisation of the Product S19

References S21

1. Definitions

Some of the terms below are inconsistently used in the literature. Consequently, a glossary of definitions is given in this section. These definitions apply for both the main text and the Supporting Information.

Continuous process/reactor

Reactants are continuously added to the system, while products are continuously removed from the system at a constant mass-flow rate. The continuous flows of components enter and leave the unit at well-defined entry and exit points. Continuous reactors are designed to operate at or near a steady state by controlling the operating conditions, which are largely the same from one day to the next. The definition is based on Perry's chemical engineers' handbook,[1] and the article by Poechlauer *et al*.[2]

Flow reactor

"A flow reactor is a type of continuous reactor where all portions of the feed stream have the same residence time, that is; mixing occurs only in the radial direction and not in the axial one."[1]

Carbon footprint

The carbon footprint is expressed by the equivalent kg of CO_2 that the manufacturing process generates during the production of 1 kg of product as shown in Equation S1:

$$\text{Carbon footprint} = \frac{\text{equivalent kg of CO}_2}{\text{kg isolated product}} \qquad \text{Eq. S1}$$

E-factor

The E-factor is defined as the mass of generated waste for 1 kg of product as shown in Equation S2:

$$\text{E} - \text{factor} = \frac{\text{kg waste generated}}{\text{kg isolated product}} \qquad \text{Eq. S2}$$

Turnover number (TON)

Turnover number equals to the moles of desired product formed over the number of active centres or surface area (heterogeneous catalyst) as shown in Equation S3:

$$\text{TON} = \text{n}_{\text{product}} \times \text{n}_{\text{catalyst}}^{-1} \qquad \text{Eq. S3}$$

Turnover frequency (TOF)

Turnover frequency equals to the desired product formed in 1 hour over the number of active centres or surface area (heterogeneous catalyst) as shown in Equation S4:

$$\mathrm{TOF} = \mathrm{TON} \times \mathrm{t}^{-1} \qquad \text{Eq. S4}$$

Solute rejection

Rejection of a compound "i" is defined as the ratio between the concentration of that compound in the permeate stream over the concentration in the retentate stream as shown in Equation S5:

$$\mathrm{R} = 100 \times \left(1 - \frac{\mathrm{C_{permeate,i}}}{\mathrm{C_{retentate,i}}}\right) = (\%) \qquad \text{Eq. S5}$$

Permeance

Permeance equals to the volume of liquid that permeates the membrane per unit of time, per membrane area and per applied pressure as shown in Equation S6:

$$\mathrm{Permeance} = \frac{\mathrm{V_{permeate}}}{\mathrm{Time} \times \mathrm{Area} \times \mathrm{Pressure}} = (\mathrm{L} \times \mathrm{m}^{-2} \times \mathrm{h}^{-1} \times \mathrm{bar}^{-1}) \qquad \text{Eq. S6}$$

Purity

Purity compares the mass of the product in the retentate stream, with the sum of masses of the compounds present in that stream as shown in Equation S7:

$$\mathrm{Purity} = 100 \times \left(\frac{\mathrm{Cretentate\ product}}{\mathrm{Cretentate\ product} + \mathrm{Cretentate\ reagent}}\right) = (\%) \qquad \text{Eq. S7}$$

Productivity

Productivity is defined as the ratio of the concentration of the pure product in the retentate stream over the volume of the reactor unit times the flow rate of the reactor's inlet stream as shown in Equation S8:

$$\mathrm{Productivity} = \frac{\mathrm{F_{feed}} \times \mathrm{C_{crude\ product}}}{\mathrm{V_{reactor}}} = (\mathrm{g} \times \mathrm{L}^{-1} \times \mathrm{h}^{-1}) \qquad \text{Eq. S8}$$

Equilibrium time

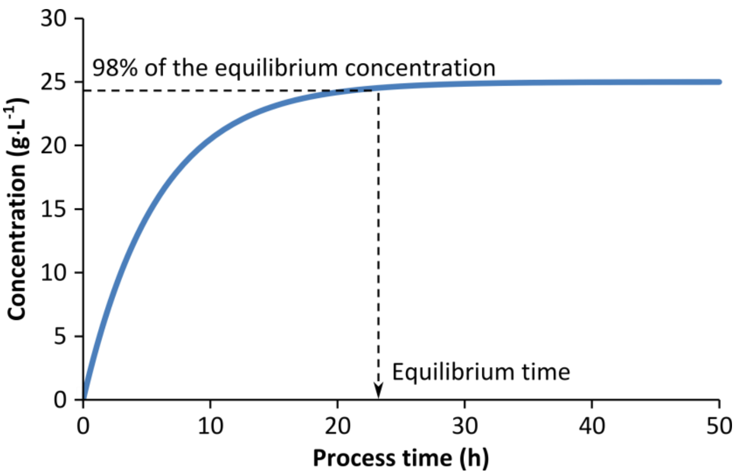

Figure S1. Equilibrium time refers to the time for a solute to reach 98% of its final concentration in either the permeate stream or the retentate stream. In the process modelling the equilibrium time was used to define the start of the steady-state operation.

Solvent recycle

The percentage of solvent that gets recycled over the amount of the total solvent that is used for 1 kg of product as shown in Equation S9:

$$\text{Solvent recycle} = 100 \times \frac{\text{kg recovered solvent}}{\text{total kg solvent used}} = (\%) \qquad \text{Eq. S9}$$

Reagent recycle

The percentage of reagent that gets recycled over the amount of the total reagent that enters the feed stream of the membrane unit (i.e. leaves the flow reactor) as shown in Equation S10:

$$\text{Reagent recycle} = 100 \times \frac{\text{kg recovered reagent}}{\text{total kg reagent}} = (\%) \qquad \text{Eq. S10}$$

Solvent consumption

The required total mass of solvent for the production of 1 kg of product as shown in Equation S11:

$$\text{Solvent consumption} = \frac{\text{kg solvent used}}{\text{kg product}} \qquad \text{Eq. S11}$$

Reaction conversion

The conversion percentage of the reaction is the molar ratio of the remaining substrate at the end of the reaction over the initial substrate amount as shown in Equation S12:

$$\text{Coversion} = 100 \times \left(1 - \frac{n_{\text{remaining substrate}}}{n_{\text{initial substrate}}}\right) = (\%) \qquad \text{Eq. S12}$$

Retentate/permeate flow rate ratio

The retentate/permeate flow rate ratio is defined as the flow rate of the retentate stream over the flow rate of the permeate stream as shown in Equation S13.

$$\text{Retentate/permeate flow rate ratio } (\lambda) = \frac{\text{flow rate of the retentate stream}}{\text{flow rate of the permeate stream}} \qquad \text{Eq. S13}$$

Membrane loop volume/area ratio

The membrane loop volume/area ratio is defined as the total volume of the membrane loop over the total membrane area in the nanofiltration unit as shown in Equation S14:

$$\text{Membrane loop volume/area ratio} = \frac{\text{Volume of the membrane loop}}{\text{Area of the membrane}} = (\mathrm{L} \times \mathrm{m}^{-2}) \qquad \text{Eq. S14}$$

2. Reaction Kinetics in the Flow Reactor

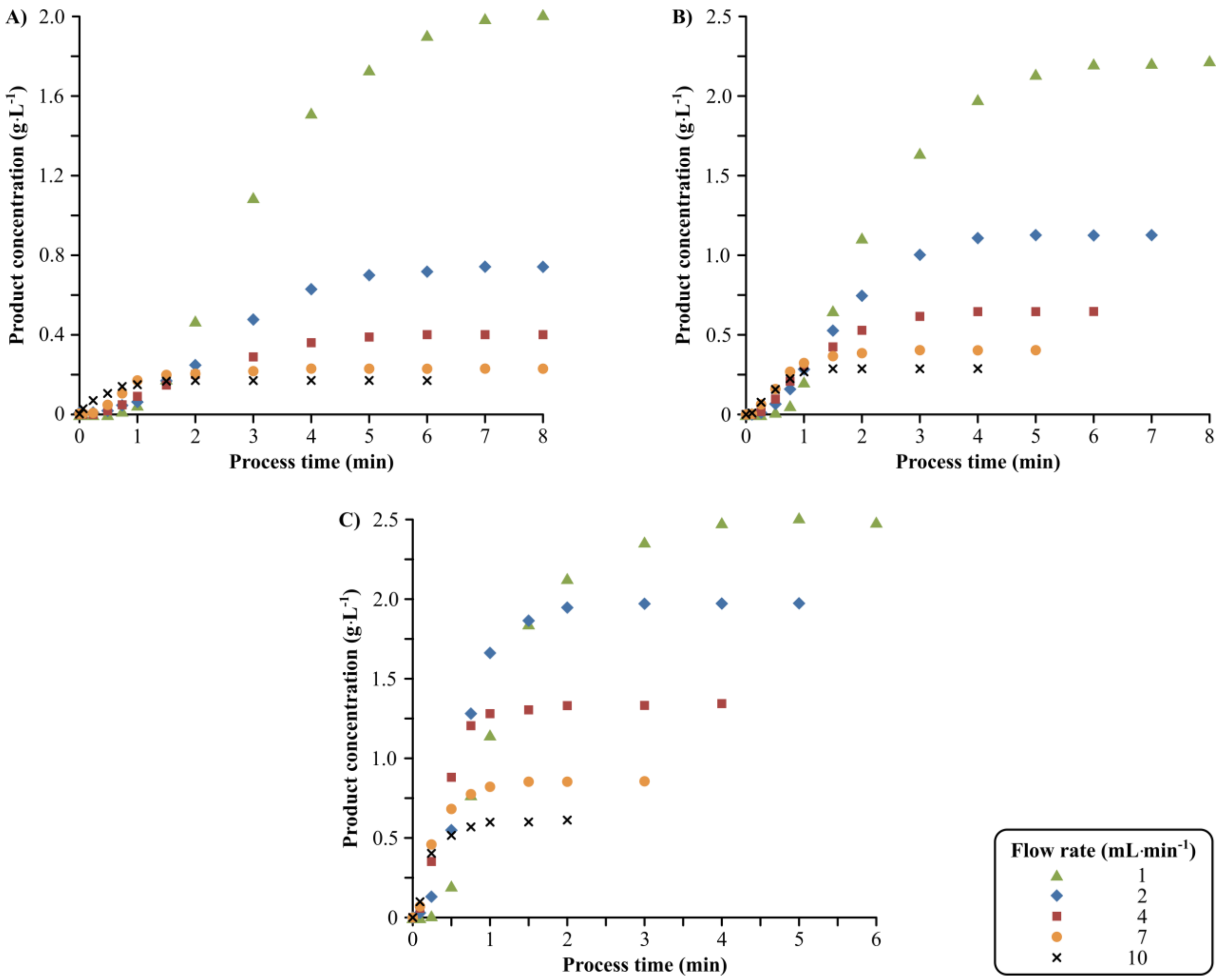

Figure S2. The effect of flow rate on the product concentration profile at various temperatures: 30 °C (**A**), 40 °C (**B**), and 50 °C (**C**). The feed solution containing 9.3 mM *trans*-chalcone substrate and 5 equivalents of nitromethane reagent in acetone was allowed to pass through the packed bed flow reactor having 8.2 grams of Amberlite™ IRA67 trialkyl amine base catalyst.

3. Process Configuration for Membrane Screening

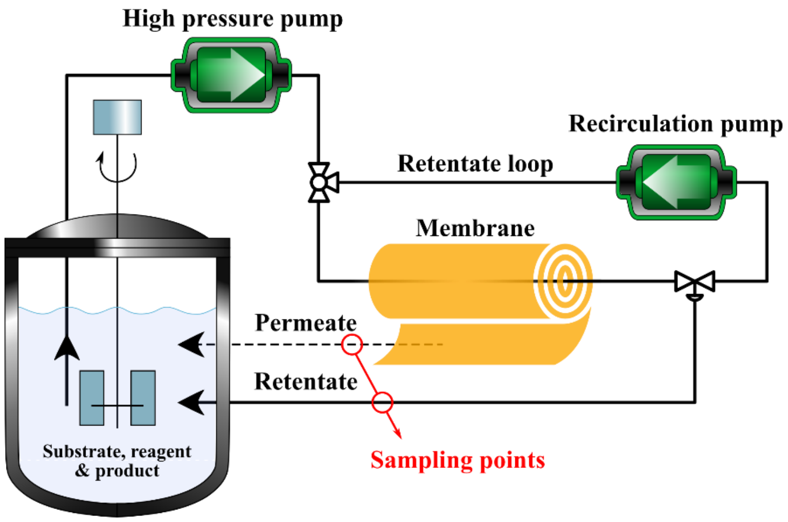

Figure S3. Schematic process configuration for membrane screening, i.e. determination of solute rejection and flux at 10-40 bar pressure using different membranes. The nanofiltration unit had 0.021 m^2 membrane area and 35 mL volume, corresponding to 1.67 $L{\cdot}m^{-2}$ membrane loop volume/area ratio. A recirculation pump set at 1 $L{\cdot}min^{-1}$ ensured homogeneous solute concentration in the membrane loop. The feed solution comprised of the product, the substrate and the reagent at 1 $g{\cdot}L^{-1}$ concentration in acetone.

4. Process Modelling

The purpose of the process modelling is to have a predictive tool for the performance of the nanofiltration-enabled separation. Based on the solute rejections and the permeability obtained through the membrane screening as well as on the system parameters a model was created in Matlab® to give solutions to the mass balances. These mass balances formed a set of two ordinary differential equations and were calculated using a numerical solution (Runge-Kutta method). The process flow sheet (see **Figure 7** in the main article) reveals that the flow reactor has one inlet and one outlet streams. Therefore they are bound to be equal in terms of flow as preservation of mass principle dictates, Equation S15:

$$\mathrm{F_{feed}^{reactor}} = \mathrm{F_{crude}} \qquad \text{Eq. S15}$$

where $\mathrm{F_{feed}^{reactor}}$ is the inlet flow to the flow reactor and $\mathrm{F_{crude}}$ is the outlet flow of the flow reactor unit. The outlet flow can also be considered as the crude product stream or the inlet flow to the nanofiltration unit. With regards to the nanofiltration unit there are three streams that are connected to it: the inlet stream ($\mathrm{F_{crude}}$), and two outlet streams, namely the retentate stream ($\mathrm{F_{retentate}}$) and the permeate stream ($\mathrm{F_{permeate}}$). As the preservation of mass principle dictates the inlet flow equals to the sum of the two outlet flows, Equation S16:

$$\mathrm{F_{crude}} = \mathrm{F_{retentate}} + F_{permeate} \qquad \text{Eq.S16}$$

Since the substrate conversion in the flow reactor was set to be 100% the nanofiltration inlet stream contains only two compounds, the product and the unreacted 4 molar equivalents of reagent. The mass balances for these two compounds in the nanofiltration unit are given in differential Equations S17-S19.

$$\frac{\mathrm{dC_{retentate}^{reagent}}}{\mathrm{dt}} = \frac{1}{\mathrm{V_{membrane}}} \times \left[\left(\mathrm{F_{crude}} \times \mathrm{C_{crude}^{reagent}}\right) - \left(\mathrm{F_{retentate}} \times \mathrm{C_{retentate}^{reagent}}\right) - \left(\mathrm{F_{permeate}} \times \left(1 - \mathrm{R_{reagent}}\right) \times \mathrm{C_{retentate}^{reagent}}\right)\right] \qquad \text{Eq. S17}$$

where $\mathrm{C_{retentate}^{reagent}}$ (g·L^{-1}) is the concentration of reagent in the retentate stream, $\mathrm{V_{membrane}}$ (L) is the volume of the membrane loop, $\mathrm{C_{crude}^{reagent}}$ (g·L^{-1}) is the concentration of the reagent in the crude product stream, i.e. nanofiltration inlet stream. Substitution of the flows in Equation S16 and the retentate/permeate flow rate ratio (λ, Equation S13) gives Equation S18. Based on the mass balances the same expression can be derived for the product as shown in Equation S19.

$$\frac{\mathrm{dC_{retentate}^{reagent}}}{\mathrm{dt}} = \frac{1}{\mathrm{V_{membrane}}} \times$$

$$\left[\left(\mathrm{F_{crude}} \times \mathrm{C_{crude}^{reagent}}\right) - \left(\frac{\mathrm{F_{crude}} \times \mathrm{C_{retentate}^{reagent}}}{1+\frac{1}{\lambda}}\right) - \left(\frac{\mathrm{F_{crude}} \times \left(1-\mathrm{R_{reagent}}\right) \times \mathrm{C_{retentate}^{reagent}}}{1+\lambda}\right)\right] \qquad \text{Eq. S18}$$

$$\frac{dC_{retentate}^{product}}{dt} = \frac{1}{V_{membrane}} \times$$

$$\left[\left(F_{crude} \times C_{crude}^{product}\right) - \left(\frac{F_{crude} \times C_{retentate}^{product}}{1+\frac{1}{\lambda}}\right) - \left(\frac{F_{crude} \times (1-R_{product}) \times C_{retentate}^{product}}{1+\lambda}\right)\right] \quad \text{Eq. S19}$$

Practically these two equations are being solved simultaneously as a system. The generated solution is a numerical one, and it is represented by curves of concentration over time. **Figure 8** in the main article shows the fitting of the experimental data with the predicted concentration profiles for both the retentate stream and the permeate stream for both compounds: the reagent and the product.

The predicted concentration profiles provide useful information about the time when the two compounds reach equilibrium (see **Figure S1**). When the equilibrium concentration is reached the system is considered to be at steady-state, and the recycle of the permeate stream can be commenced. The permeate reaches steady state faster than the retentate, which can be explained by the differences in rejections of the corresponding two compounds for a given retentate/permeate flow rate ratio value. The retentate/permeate flow rate ratio for the continuous hybrid process was set at 0.1, i.e. the flow rate of the retentate stream is 10 times smaller than the flow rate of the permeate stream. Starting from 0 $g \cdot L^{-1}$ concentration in the nanofiltration, it will take about 2.5 hours for the reagent to fill the nanofiltration unit and reach equilibrium concentration. On the other hand, the equilibrium concentration of the product is about 10 times higher than that of the reagent, and consequently it will take significantly more time to reach equilibrium (30 hours). In order to minimise the start-up time and the solvent consumption, the recycle of the permeate stream was commenced when the permeate concentration of the reagent reaches equilibrium.

Once the permeate stream is connected to the inlet of the flow reactor the $F_{feed}^{reactor}$ needs to be adjusted in order to maintain undisturbed, constant flow rate and concentrations entering the flow reactor. Again the resolved mass balances give the solution. For the two compounds that enter the reactor, namely substrate and reagent, Equation S20 describes the necessary flow rate of the feed of the reactor, and two equations to describe the concentration of reagent (Equation S21) and the substrate (Equation S22).

$$F_{feed}^{reactor} = F_{permeate} + F_{pre-feed}^{reactor} \quad \text{Eq. S20}$$

$$F_{feed}^{reactor} \times C_{feed}^{reagent} = F_{permeate} \times C_{permeate}^{reagent} + F_{pre-feed}^{reactor} \times C_{pre-feed}^{reagent} \quad \text{Eq. S21}$$

$$F_{feed}^{reactor} \times C_{feed}^{substrate} = F_{pre-feed}^{reactor} \times C_{pre-feed}^{substrate} \quad \text{Eq. S22}$$

where $C_{feed}^{substrate}$ and $C_{feed}^{reagent}$ are the concentrations of the substrate and the reagent in $F_{feed}^{reactor}$, $C_{pre-feed}^{substrate}$ and $C_{pre-feed}^{reagent}$ are the concentrations of the substrate and the reagent in $F_{pre-feed}^{reactor}$, and $C_{permeate}^{reagent}$ is the equilibrium concentration of the reagent in the permeate stream.

5. Sensitivity analysis for the hybrid process

Table S1. The variation of five system parameter settings (conversion, product and reagent rejections, retentate/permeate flow rate ratio, and membrane loop volume/area) and their effects on four process metrics (solvent consumption, product purity, productivity and equilibrium time). The arrows indicate the increase or decrease in the process metrics. The analysis was carried out using one-factor-at-a-time variation. The concentration profiles for each simulation are shown in Figure S4-S10.

System Parameter		Solvent Consumption ($kg \cdot kg^{-1}$)	Purity (%)	Productivity ($g \cdot L^{-1} \cdot h^{-1}$)	Equilibrium Time (h)
**Membrane loop volume/area ($L \cdot m^{-2}$)**	0.24			15	0.4
	0.95			6	1.5
	1.67*	29	91.5	3.8	2.5
	2.86			2.3	4.4
	4.29			1.6	6.6
**Product Rejection (%)**	90	58	84.4	1.9	13
	95	43	87.8	2.5	17
	98	35	90.0	3.1	21
	99	32	90.8	3.4	23
	100*	29	91.5	3.8	2.5
**Reagent Rejection (%)**	5		92.0		2.4
	12*		91.5		2.5
	20	29	90.8	3.8	2.8
	30		89.8		3.1
	45		87.8		3.9
**Conversion (%)**	80	36.0	84.5	3.0	
	90	32.0	88.3	3.4	
	96	30.0	90.2	3.6	2.5
	98	29.4	90.9	3.7	
	100*	28.8	91.5	3.8	
**Retentate/permeate flow rate ratio, λ (–)**	0.05	15	95.3		
	0.07	21	93.7		
	0.1*	29	91.5	3.8	2.5
	0.15	41	88.3		
	0.25	63	83.3		

*The actual parameter values applied during the continuous hybrid process (Figure 8 in the main article).

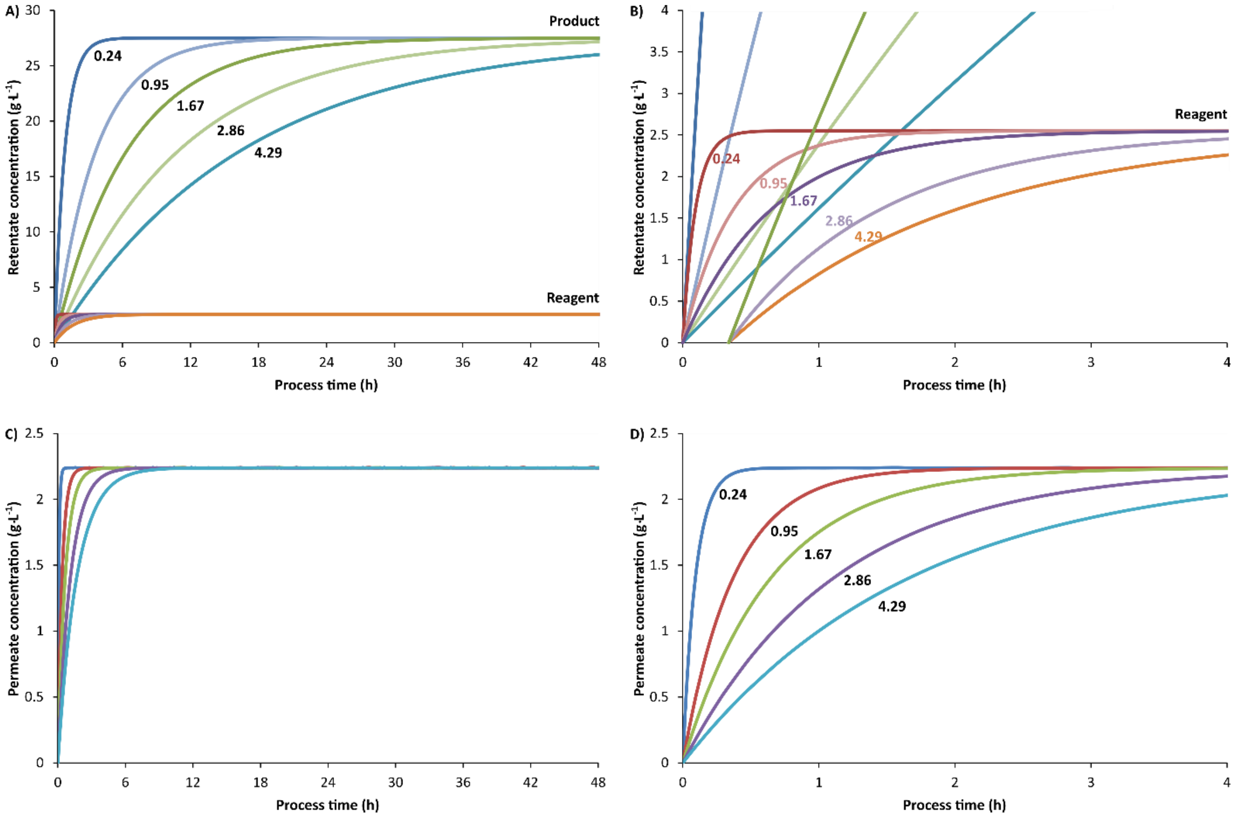

Figure S4. The concentration profiles for the retentate stream (**A**-**B**) and the permeate stream (**C**-**D**) while varying the membrane loop volume/area ratio. Panels **B** and **D** are magnifications of panels **A** and **C** in order to reveal the effect of the membrane loop volume/area ratio on the concentration profile during the start-up of the process. The 0.24-4.39 values on each concentration curve indicate the different membrane loop volume/area ratios expressed in L·m^{-2}. The membrane area was kept constant at 0.021 m^2 and the membrane loop volume was varied at 5, 20, 35, 60, 90 mL values, corresponding to 0.24, 0.95, 1.67, 2.86, 4.29 L·m^{-2}. The actual value for the membrane loop volume/area ratio during the hybrid process in the main manuscript was 1.67 L·m^{-2}. See Table S1 as well as Figure 9A in the main manuscript for the effect of the membrane loop volume/area ratio on the product purity, productivity, solvent consumption and equilibrium time.

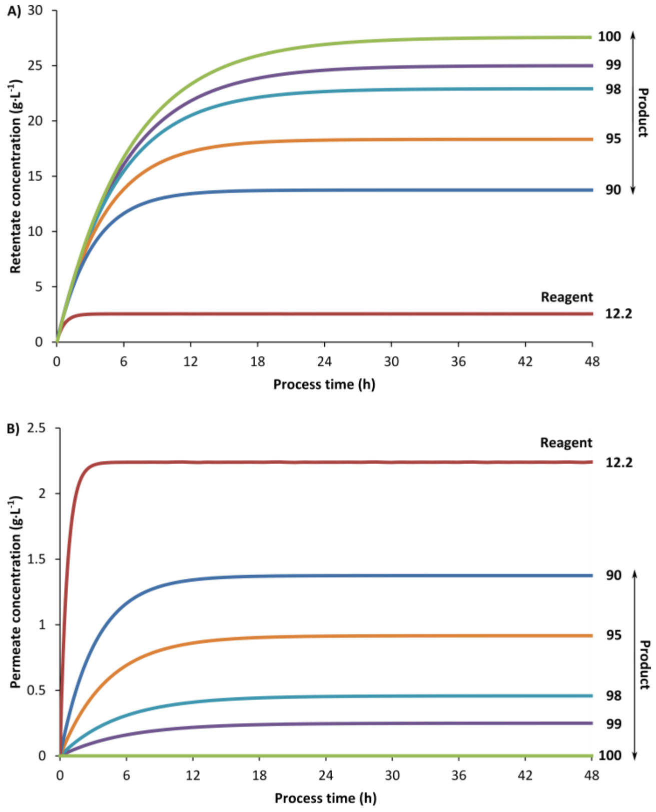

Figure S5. The concentration profiles for the retentate stream (**A**) and the permeate stream (**B**) while varying the product rejection. The values next to the concentration curves are the rejections, which was kept constant for the reagent (12.2%) and varied between 90% and 100% for the product. The actual value for the reagent rejection during the hybrid process in the main manuscript was 12.2%. See Table S1 as well as Figure 9B in the main manuscript for the effect of the product rejection on the product purity, productivity, solvent consumption and equilibrium time.

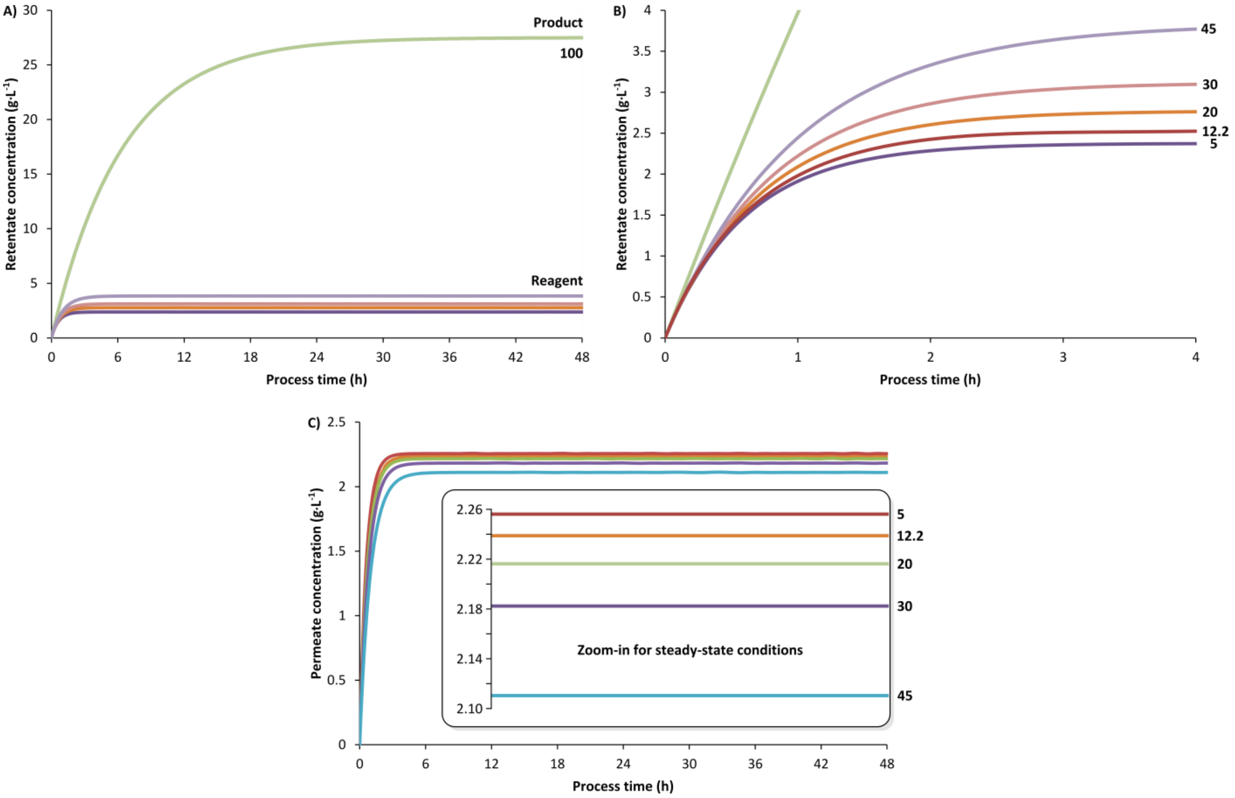

Figure S6. The concentration profiles for the retentate stream (**A**-**B**) and the permeate stream (**C**) while varying the reagent rejection. Panel **B** is a magnification of panel **A** in order to reveal the effect of the reagent rejection on the concentration profile during the start-up of the process. The values next to the concentration curves are the rejections, which was kept constant for the product (100%) and varied between 5% and 45% for the reagent. The actual value for the product rejection during the hybrid process in the main manuscript was 100%. See Table S1 as well as Figure 9C in the main manuscript for the effect of the reagent rejection on the product purity, productivity, solvent consumption and equilibrium time.

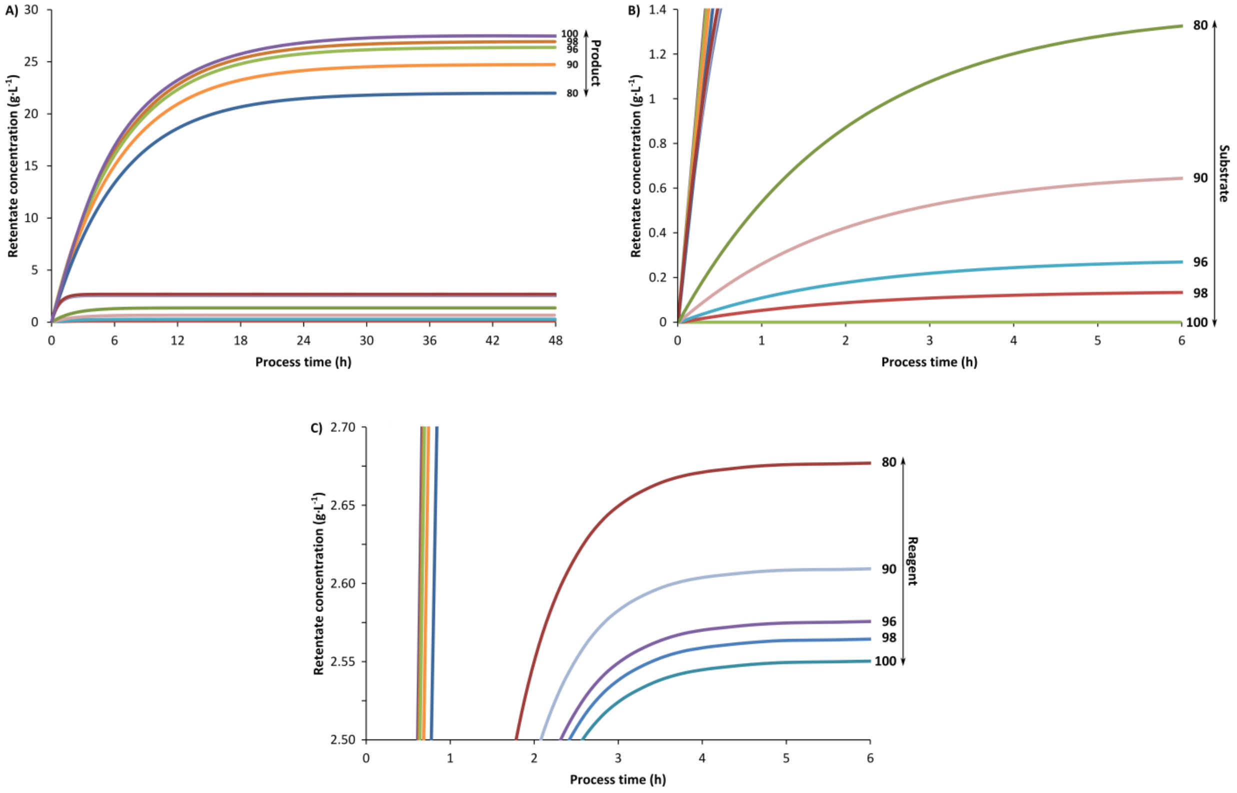

Figure S7. The concentration profiles for the retentate stream while varying the substrate conversion. The values next to the concentration curves are the conversions from 80% to 100%. Panel **A**, **B** and **C** show the concentration profile for the product, the substrate and the reagent, respectively. The rejection values used for the simulation were determined during the membrane screening: 12.2% for the reagent, 78.6% for the substrate and 100% for the product. See Table S1 as well as Figure 9D in the main manuscript for the effect of the conversion on the product purity, productivity, solvent consumption and equilibrium time.

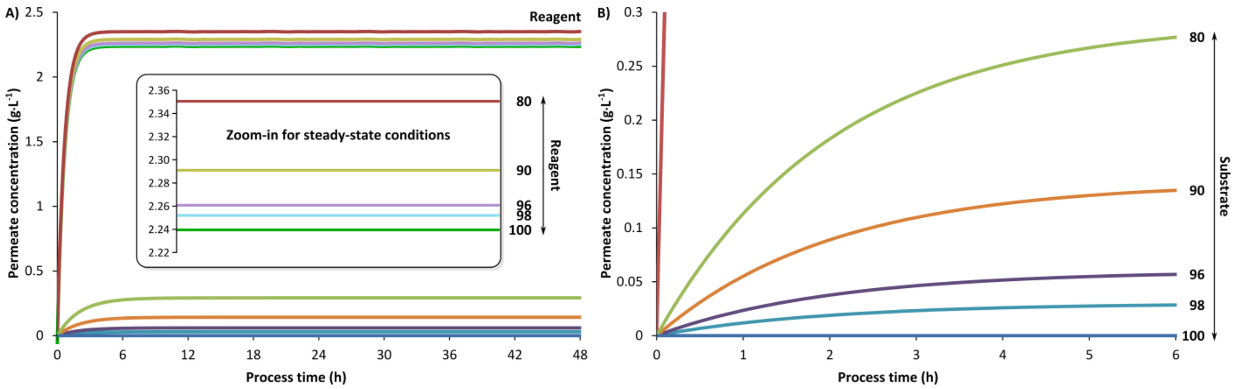

Figure S8. The concentration profiles for the permeate stream while varying the substrate conversion. The values next to the concentration curves are the conversions from 80% to 100%. Panel **A** and **B** show the concentration profile for the reagent and the substrate, respectively. The rejection values used for the simulation were determined during the membrane screening: 12.2% for the reagent and 78.6% for the substrate. The product rejection was 100% and consequently the product is not present in the permeate stream.

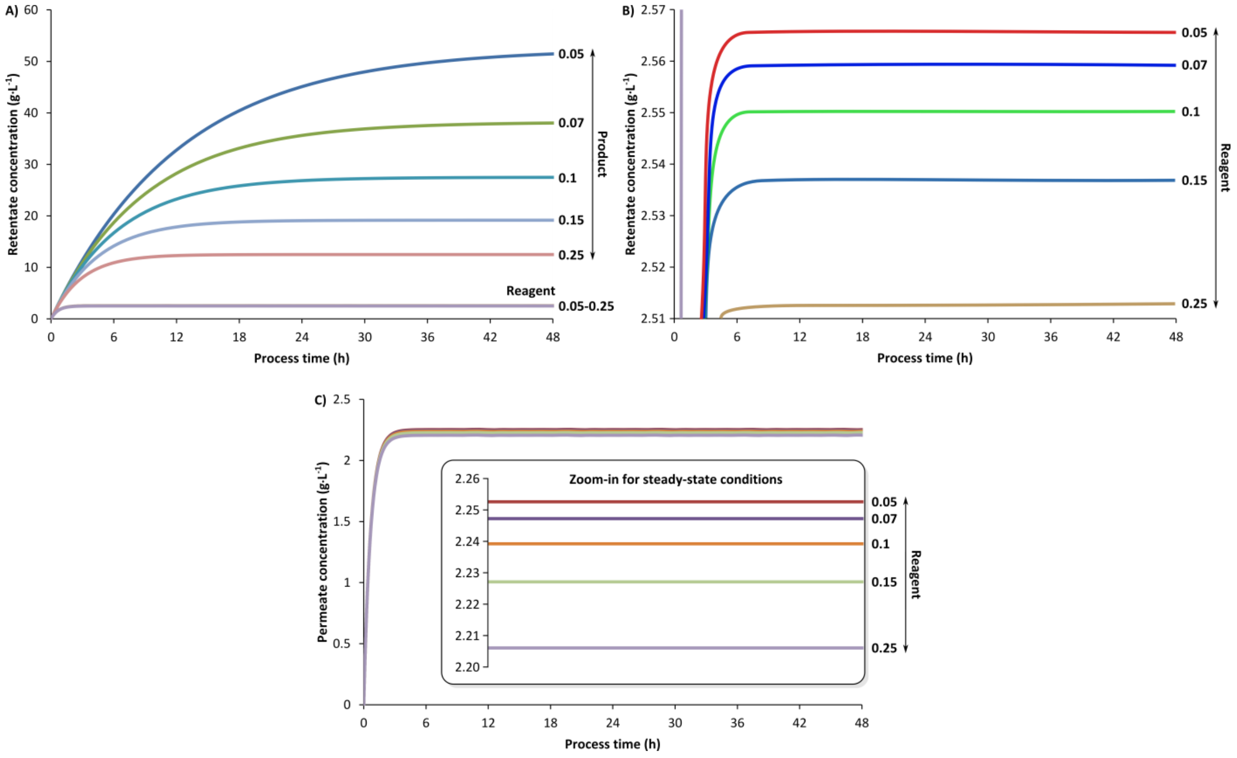

Figure S9. The concentration profiles for the retentate stream (**A**-**B**) and the permeate stream (**C**) while varying the retentate/permeate flow rate ratio. Panel **B** is a magnification of panel **A** in order to reveal the effect of the retentate/permeate flow rate ratio on the concentration profile. The values next to the concentration curves are the retentate/permeate flow rate ratio values varied between 0.05 and 0.25. The actual value for the retentate/permeate flow rate ratio during the hybrid process in the main manuscript was 0.1. See Table S1 as well as Figure 9E in the main manuscript for the effect of the retentate/permeate flow rate ratio on the product purity, productivity, solvent consumption and equilibrium time.

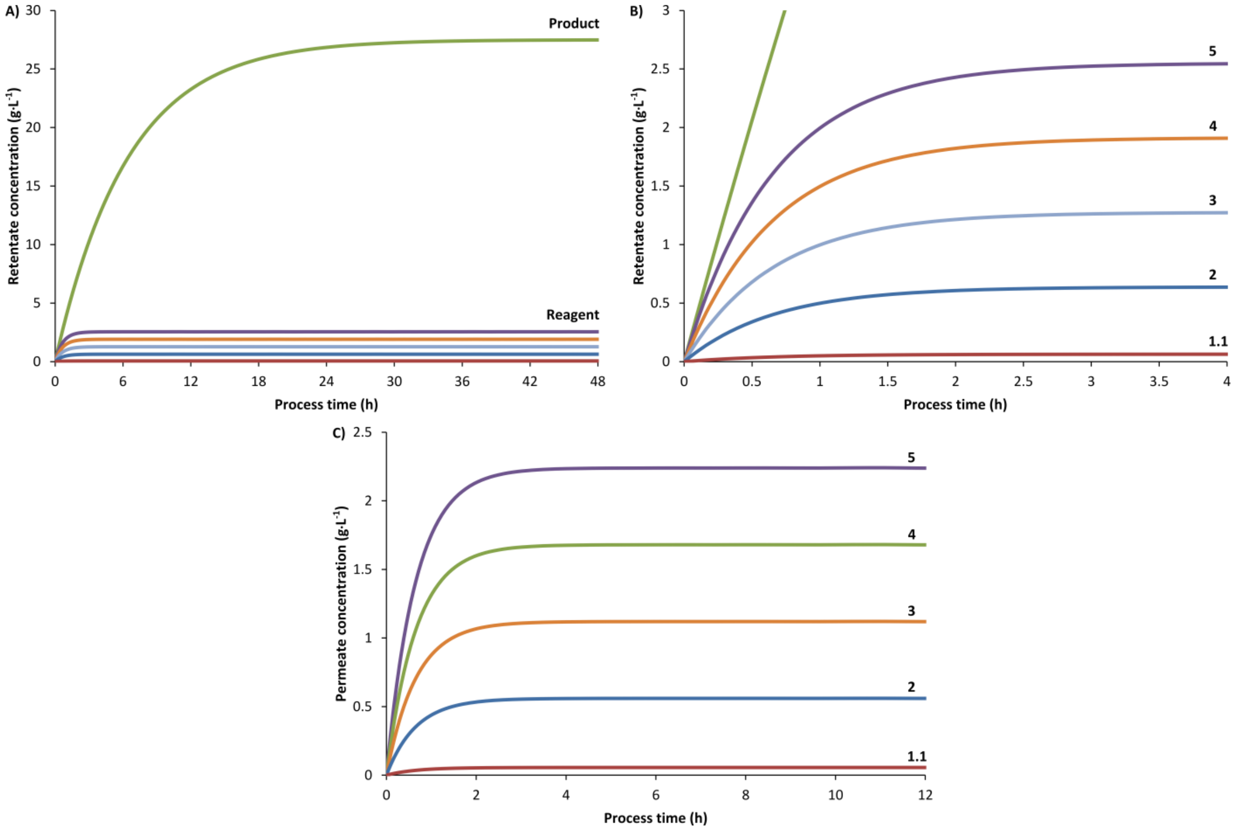

Figure S10. The concentration profiles for the retentate stream (**A**-**B**) and the permeate stream (**C**) while varying the excess of reagent. Panel **B** is a magnification of panel **A** in order to reveal the effect of the excess of reagent on the concentration profile. The values on the concentration curves are the reagent:substrate molar equivalents representing the excess of reagent. The actual value for the reagent:substrate molar equivalent for the hybrid process in the main manuscript was 5. See Figure 9F in the main manuscript for the effect of the excess of reagent on the product purity while the reagent rejection is varied.

6. Green metrics analysis

Table S2. Summary of the values of energy consumption (kWh), waste generation (kg) and equivalents of CO_2 (kg) per kg of product. In the absence of the nanofiltration unit both the solvent and the excess reagent are disposed via incineration. The highlighted cells indicate the most significant contributors to the above measures. The percentage shows their contribution to the overall assessment.

	without *in situ* solvent & reagent recovery			with *in situ* solvent & reagent recovery		
	**Energy consumption** (kWh·kg^{-1})	**Waste generation** (kg·kg^{-1})	**Carbon footprint** (kg·kg^{-1})	**Energy consumption** (kWh·kg^{-1})	**Waste generation** (kg·kg^{-1})	**Carbon footprint** (kg·kg^{-1})
**High pressure pump**	0.0117	n.a.	0.0080	0.4678	n.a.	0.32
**Recirculation pump**	n.a.*	n.a.	n.a.	12.3	n.a.	8.44
**Thermostat (50°C)**	3300 (99.99%)	n.a.	2257 (78.22%)	3300 (99.61%)	n.a.	2257 (97.16%)
**Solvent**	n.a.	316.4 (99.68%)	626.47 (21.7%)	n.a.	28.8 (99.27%)	56.95 (2.45%)
**Reagent**	n.a.	0.91	1.80	n.a.	0.09	0.184
**Catalyst**	n.a.	0.1175	0.117451	n.a.	0.1175	0.117451
**Membrane module**	n.a.	n.a.	n.a.	n.a.	0.00019504	0.0065039
**Total**	**3300**	**913.42**	**2885.6**	**3312.8**	**28.97**	**2323.2**

*n.a. not applicable

The energy consumption of the thermostat of the jacketed flow reactor was measured over an hour with a Fluke 1736 power logger with resolution 10 mA and accuracy ±0.1%, then it was multiplied by the operation time of the process in order to determine the total energy consumption of the device (Equation S23). The same methodology was followed in order to determine the energy consumption of the high pressure and the recirculation pumps (Equation S24).

$$\text{Energy (kWh)} = \text{Power(kW)} \times \text{time(h)} \qquad \text{Eq. S23}$$

$$\text{Power (kW)} = \frac{\text{Flow rate }(\text{L}\cdot\text{min}^{-1})\times\text{pressuredifference (bar)}}{600} \qquad \text{Eq. S24}$$

For the calculation of the carbon footprint, the energy used for the equipment and the waste generated were converted to equivalent CO_2. The values used were obtained from Ecoinvent® database.[3] For the energy source, the low voltage (AC-240 V) electrical energy provided by the UK national grid was selected. The equivalent CO_2 for the incineration of the chemical waste (reagent, solvent, catalyst, membrane) was also obtained from Ecoinvent® database.[1] The calculation took into account the carbon content of the catalyst,[4] and the membrane module.[5] For both of processes — with and without the *in situ* solvent and reagent recycle — the majority of the CO_2 emissions derive from the solvent waste and the thermostat.

This paragraph explains the derivation of the carbon footprint versus processing time plot shown on Figure 10C in the main text. For the process without *in situ* recovery of the solvent and reagent, the concentration of the product that leaves the reactor is constant (2.5 $g{\cdot}L^{-1}$). Therefore the amount of the solvent needed for collecting 1 kg of product will remain constant (316.4 $g{\cdot}L^{-1}$) throughout the process. In other words, the solvent consumption (400 $L{\cdot}kg^{-1}$) and the carbon footprint (2885.6 $kg{\cdot}kg^{-1}$) are constant over time. On the other hand, the process with *in situ* recovery of the solvent and reagent requires an initial ~24 h until the nanofiltration unit reaches steady state (see Figure 8 in the main text). During this period, the concentration of the product in the retentate stream exponentially increases from zero until steady state (27.5 $g{\cdot}L^{-1}$) is reached. For low concentrations of product in the retentate stream, the relative solvent consumption is large per kg of product, translating to high carbon footprint. As the product concentration in the retentate stream increases, the solvent consumption for 1 kg of product decreases along with the carbon footprint. At steady state the product has a higher concentration (27.5 $g{\cdot}L^{-1}$) than the product in the reactor outlet (2.5 $g{\cdot}L^{-1}$) and hence the solvent consumption for 1 kg of product becomes 91% less than without the *in situ* recovery, and consequently leads to a reduced carbon footprint of 19% (see Figure 10C in the main text).

7. Chemical characterisation of the product

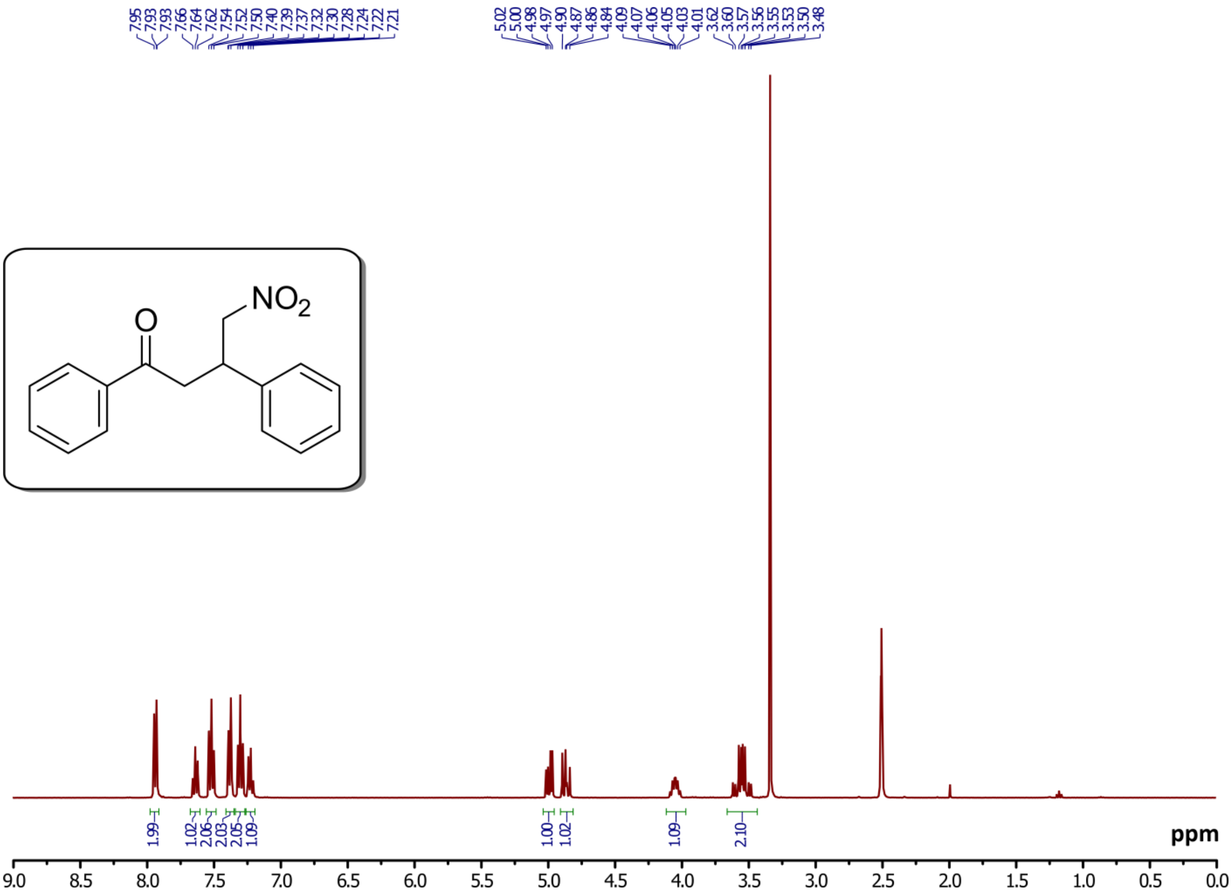

Figure S11. ^{1}H-NMR of 4-nitro-1,3-diphenylbutan-1-one product. ^{1}H-NMR (400 MHz, DMSO-d_6) δ= 3.48–3.62 (m, 2H, $COCH_2$), 4.01–4.09 (m, 1H, CH), 4.84–4.90 (m, 1H, CH_2NO_2), 4.97–5.02 (m, 1H, CH_2NO_2), 7.22 (t, 1H, ArCH-4, *J*=7.6 Hz), 7.30 (t, 2H, ArCH-3 and 5, *J*=7.6 Hz), 7.38 (d, 2H, ArCH-2 and 6, *J*=7.2 Hz), 7.52 (t, 2H, ArCH-3' and 5', *J*=7.6 Hz), 7.64 (t, 1H, ArCH-4', *J*=7.6 Hz), 7.94 (d, 2H, ArCH-2' and 6', *J*=7.2 Hz) ppm.

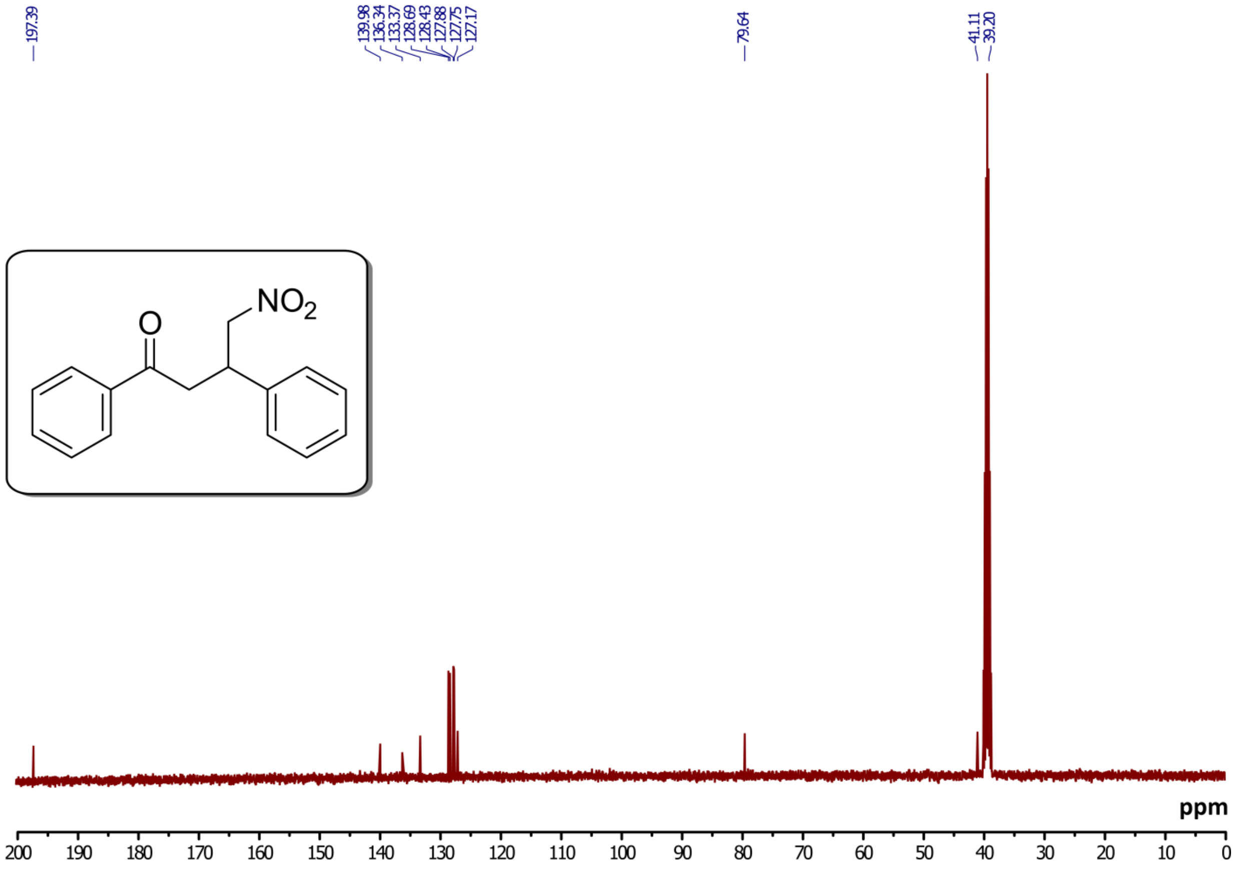

Figure S12. ^{13}C-NMR of 4-nitro-1,3-diphenylbutan-1-one product. ^{13}C-NMR (400 MHz, DMSO-d_6) δ= 39.20 (CH), 41.11 (CH_2CO), 79.64 (CH_2NO_2), 127.17 (ArC-4), 127.75 (ArC-2 and 6), 127.88 (ArC-3 and 5), 128.43 (ArC-3’ and 5’), 128.69 (ArC-2’ and 6’), 133.37 (ArC-4’), 136.34 (ArC-1’), 139.98 (ArC-1), 197.39 (C=O) ppm.

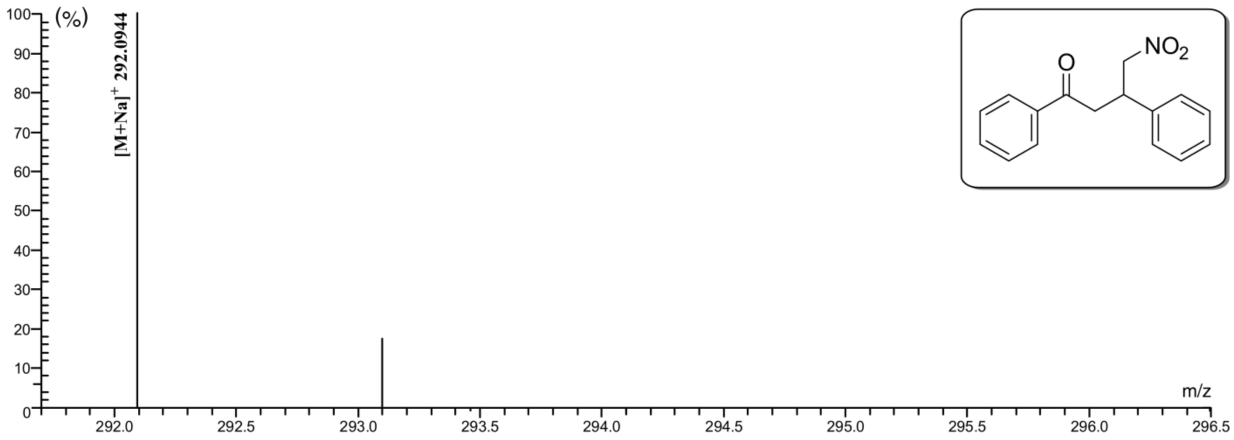

Figure S13. Mass spectrum of 4-nitro-1,3-diphenylbutan-1-one product. MS: 292.0934 ($M+Na^+$)

References

1 R.H. Perry, D.W. Green, Perry's chemical engineers' handbook. 8th Edition, McGraw-Hill Professional, **2007**

2 P. Poechlauer, J. Manley, R. Broxterman, B. Gregertsen, M. Ridemark, Continuous processing in the manufacture of active pharmaceutical ingredients and finished dosage forms: an industry perspective, *Org. Process Res. Dev.*, **2012**, *16*, 1586-1590.

3 Ecoinvent v2.0 database. Swiss Centre for Life Cycle Inventories, Dübendorf, Switzerland, can be found under: http://www.ecoinvent.org.

4 A. G. Firth, K. Wilson, C. G. Baumann, I. J. S. Fairlamb, *Nucleosides, Nucleotides and Nucleic Acids*, **2011**, *30*, 168-184.

5 W. Lawler, Z. Bradford-Hartke, M. J. Cran, M. Duke, G. Leslie, B. P. Ladewig, P. Le-Clech, *Desalination*, **2012**, *299*, 103-112.
